# CHAF1A Blocks Neuronal Differentiation and Promotes Neuroblastoma Oncogenesis via Metabolic Reprogramming

**DOI:** 10.1002/advs.202005047

**Published:** 2021-08-08

**Authors:** Ling Tao, Myrthala Moreno‐Smith, Rodrigo Ibarra‐García‐Padilla, Giorgio Milazzo, Nathan A. Drolet, Blanca E. Hernandez, Young S. Oh, Ivanshi Patel, Jean J. Kim, Barry Zorman, Tajhal Patel, Abu Hena Mostafa Kamal, Yanling Zhao, John Hicks, Sanjeev A. Vasudevan, Nagireddy Putluri, Cristian Coarfa, Pavel Sumazin, Giovanni Perini, Ronald J. Parchem, Rosa A. Uribe, Eveline Barbieri

**Affiliations:** ^1^ Department of Pediatrics Section of Hematology‐Oncology Texas Children's Cancer and Hematology Centers Baylor College of Medicine Houston TX 77030 USA; ^2^ Dan L Duncan Comprehensive Cancer Center Baylor College of Medicine Houston TX 77030 USA; ^3^ Department of BioSciences Rice University Houston TX 77005 USA; ^4^ Department of Pharmacy and Biotechnology University of Bologna Bologna 40126 Italy; ^5^ Stem Cells and Regenerative Medicine Center Center for Cell and Gene Therapy Baylor College of Medicine Houston TX 77030 USA; ^6^ Department of Molecular and Cellular Biology Baylor College of Medicine Houston TX 77030 USA; ^7^ Advanced Technology Core Baylor College of Medicine Houston TX 77030 USA; ^8^ Department of Pathology and Immunology Texas Children’s Hospital, Baylor College of Medicine Houston TX 77030 USA; ^9^ Division of Pediatric Surgery Michael E. DeBakey Department of Surgery Baylor College of Medicine Houston TX 77030 USA; ^10^ Center for Precision Environmental Health Baylor College of Medicine Houston TX 77030 USA; ^11^ Program in Developmental Biology Baylor College of Medicine Houston TX 77030 USA

**Keywords:** CHAF1A, metabolism, neural crest differentiation, neuroblastoma

## Abstract

Neuroblastoma (NB) arises from oncogenic disruption of neural crest (NC) differentiation. Treatment with retinoic acid (RA) to induce differentiation has improved survival in some NB patients, but not all patients respond, and most NBs eventually develop resistance to RA. Loss of the chromatin modifier chromatin assembly factor 1 subunit p150 (CHAF1A) promotes NB cell differentiation; however, the mechanism by which CHAF1A drives NB oncogenesis has remained unexplored. This study shows that CHAF1A gain‐of‐function supports cell malignancy, blocks neuronal differentiation in three models (zebrafish NC, human NC, and human NB), and promotes NB oncogenesis. Mechanistically, CHAF1A upregulates polyamine metabolism, which blocks neuronal differentiation and promotes cell cycle progression. Targeting polyamine synthesis promotes NB differentiation and enhances the anti‐tumor activity of RA. The authors' results provide insight into the mechanisms that drive NB oncogenesis and suggest a rapidly translatable therapeutic approach (DFMO plus RA) to enhance the clinical efficacy of differentiation therapy in NB patients.

## Graphical abstract

This manuscript utilizes three different models to demonstrate that the chromatin assembly factor 1 subunit p150 (CHAF1A) blocks neuronal differentiation and promotes neuroblastoma oncogenesis. CHAF1A functions in part by reprogramming cell metabolism and activating polyamine synthesis. Blocking polyamine synthesis is a clinically translatable approach to improve retinoic acid‐based differentiation therapy.

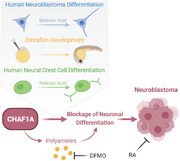

## 1 Introduction

Neuroblastoma (NB) oncogenesis is caused by disruption of neural crest (NC) development.^[^ ^1^ ^]^ During embryonic development, a population of stem cells at the neural plate border are induced to become neural crest cells (NCCs), which are multipotent stem cells derived from the primitive ectoderm.^[^ ^2^ ^]^ NCCs undergo specification, epithelial‐to‐mesenchymal transition, migration, and eventually differentiation into neurons and glia of the peripheral nervous system, facial cartilage and bone, and melanocytes.^[^ ^3^ ^]^ Many diseases are attributed to errors in NC development, such as congenital anomalies and several cancer types, most notably NB.^[^ ^4^ ^]^ Studies of transgenic mouse and zebrafish models indicate that a block in NC differentiation leads to malignant transformation of neuro‐ectodermal precursors.^[^ ^5^, ^6^ ^]^ Little is known, however, about the mechanisms that drive the oncogenic loss of differentiation in NCCs.

The biological behavior of NB is highly variable, ranging from a self‐regressing proliferation of primitive neuroblasts in infants to high‐risk metastatic disease in older children (≥ 18 months).^[^ ^7^ ^]^ Clinically, undifferentiated NB histology is an independent predictor of poor patient outcome.^[^ ^8^ ^]^ Efforts to define the mechanism by which NCC differentiation is blocked in NB have led to treatment strategies that induce differentiation with agents such as 13‐*cis*‐retinoic acid (13‐*cis*‐RA).^[^ ^9^, ^10^ ^]^ Treatment with 13‐*cis*‐RA after intensive multimodal therapy resulted in modest but consistent improvement of patient survival.^[^ ^11^, ^12^ ^]^ However, not all patients respond to 13‐*cis*‐RA therapy and most eventually develop resistance.^[^ ^13^, ^14^ ^]^

The Chromatin Assembly Factor 1 (CAF‐1) is a nuclear complex composed of the subunits p150 (CHAF1A), p60 (CHAF1B), and p48. CAF‐1 functions as a histone chaperone, controlling nucleosome assembly,^[^ ^15^ ^]^ heterochromatin maintenance,^[^ ^16^ ^]^ and DNA repair.^[^ ^17^ ^]^ CHAF1A, the primary subunit of CAF‐1, plays a central role in CAF‐1 function by interacting with multiple factors, including the heterochromatin reader protein HP1.^[^ ^18^ ^]^ CHAF1A directly interacts with the methyl‐CpG‐binding domain protein 1 (MBD1) and HP1 to initiate a gene‐silencing program via DNA methylation.^[^ ^19^ ^]^ CHAF1A is essential for normal embryonic development^[^ ^20^ ^]^ and loss of CHAF1A in homozygous mutants leads to developmental arrest in mouse and *Drosophila* models.^[^ ^21,22^ ^]^ In addition, high CHAF1A expression is associated with cell growth in breast cancer,^[^ ^23^ ^]^ deregulation of DNA repair in squamous cell carcinoma,^[^ ^24^ ^]^ and increased cell motility and invasion in Src‐transformed epithelial cells.^[^ ^25^ ^]^ In NB, we demonstrated that loss of CHAF1A function promotes NB cell differentiation and blocks tumor growth.^[^ ^26^ ^]^ However, the mechanism by which CHAF1A supports NB oncogenesis has so far remained unexplored.

Here, we show that CHAF1A restricts neuronal differentiation in zebrafish NC, human NC, and human NB, and is required for NB cell malignancy and oncogenesis. Mechanistically, CHAF1A expression alters neuronal development, cell proliferation, differentiation, and metabolism gene expression programs. Specifically, CHAF1A activates polyamine metabolism to block NB differentiation and support cell growth. Blocking polyamine synthesis restores RA‐mediated neuronal differentiation and enhances cell sensitivity to RA, suggesting that targeting polyamine metabolism is a potential rapidly translatable approach to enhance the clinical efficacy of RA in NB patients.

## 2 Results

### 2.1 CHAF1A Promotes NB Cell Malignancy and Tumorigenesis

**Figure 1:**
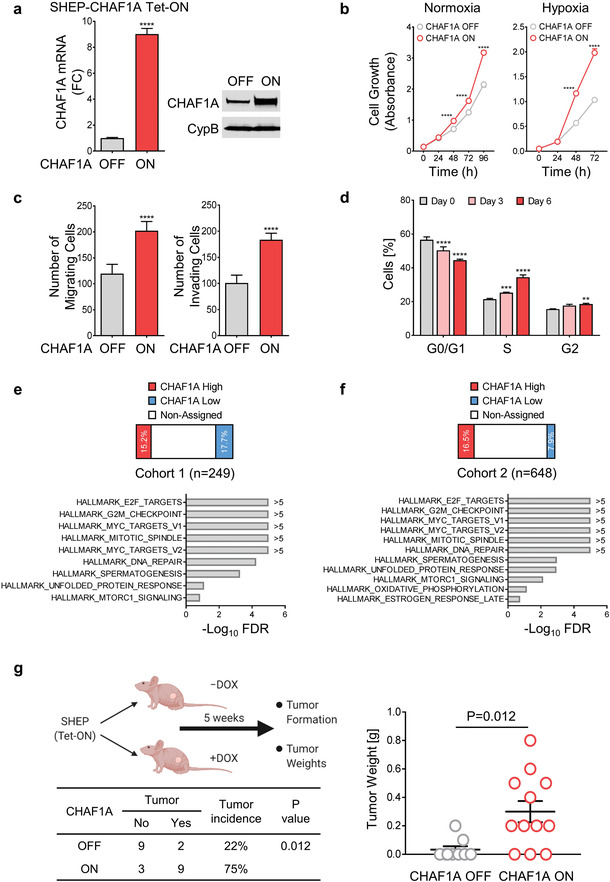
CHAF1A promotes NB aggressiveness. a) CHAF1A is turned on upon DOX induction (1 µg mL^−1^ for 72 h) in SHEP cells. Validation of CHAF1A overexpression by qPCR and western blotting is shown. Date are mean ± SD (*n* = 3); ****p < 0.0001; two‐sided unpaired *t*‐test. b) Proliferation assay in SHEP‐CHAF1A cells. Cells were cultured in normoxic and hypoxic (1% O_2_) conditions for 0–96 h. Cell number was assessed by Cell Counting Kit‐8 and are indicated by absorbance (450 nm). Mean ± SD (*n* = 4); ****p < 0.0001; two‐way ANOVA with Sidak's multiple comparisons test. c) Migration and invasion analyses of SHEP‐CHAF1A cells upon induction of CHAF1A (48 and 72 h). Mean ± SD (*n* = 5–10); ****p < 0.0001; two‐sided unpaired *t*‐test. d) Cell cycle analysis of SHEP‐CHAF1A cells upon induction of CHAF1A (3 and 6 days). Mean ± SD (*n* = 4); **p < 0.01, ***p < 0.001, ****p < 0.0001; two‐way ANOVA with Dunnett's multiple comparisons test. e,f) GSEA Hallmark analysis in patients with high and low *CHAF1A* expression in two independent patient cohorts. False discovery rate (FDR) is computed using a Benjamini–Hochberg corrected two‐sided homoscedastic *t*‐test. Pathways are ranked by −Log_10_ FDR (FDR < 0.25). g) Tumor formation upon activation of CHAF1A in an orthotopic mouse model. Low‐tumorigenic NB SHEP cells were injected into the renal capsule of NCr nude mice. Four‐week‐old mice were treated with control (*n* = 11) or DOX‐containing diet (0.625 g kg^−1^, *n* = 12) for five weeks. Tumor incidence and tumor weights are shown. Data are the mean ± SEM; comparison of tumor incidence between CHAF1A OFF and CHAF1A ON mice was computed by two‐sided Fisher's exact test, *p* = 0.012.

We previously showed that high CHAF1A expression independently predicts poor outcome in patients with NB, and that loss of CHAF1A function promotes NB cell differentiation in vivo.^[^ ^26^ ^]^ To determine how CHAF1A expression alters the NB phenotype, we expressed CHAF1A in SHEP cells using a Tet‐ON conditional system (**Figure** **1a**). Wild‐type SHEP cells have low CHAF1A protein levels compared with other NB cell lines (Figure S1, Supporting Information). Ectopic CHAF1A expression in SHEP cells increased proliferation in both normoxic and hypoxic (1% O_2_) conditions (*p* < 0.0001); however, the effect was greater in hypoxic conditions (*p* < 0.0001, Figure 1b). Moreover, CHAF1A expression significantly enhanced SHEP cell migration and invasion (*p* < 0.0001, Figure 1c). Differentiation is comprised of cell cycle arrest and implementation of lineage‐specific gene programs. Ectopic CHAF1A expression in SHEP cells promoted cell cycle progression, increasing the percentage of S‐phase cells (*p* < 0.001) and reducing the percentage of G0/G1‐phase cells (*p* < 0.0001, Figure 1d). We observed the same phenotypic changes in two additional NB cell lines, GIMEN (non MYCN‐amplified, non MNA) and NGP (MYCN‐amplified, MNA) upon ectopic CHAF1A overexpression (Figure S2a–h, Supporting Information).

To uncover CHAF1A function in NB patients, we stratified two independent patient cohorts (cohort 1 [*n* = 249] and cohort 2 [*n* = 648]^[^ ^27^ ^]^) into low and high *CHAF1A* expression groups based on average (± one standard deviation) *CHAF1A* mRNA expression. Gene set enrichment analysis (GSEA) revealed that genes in several cell cycle‐related pathways (cell cycle phase, cell cycle progress, and mitotic cell cycle, FDR < 0.25) were differentially expressed between patients with high and low *CHAF1A* expression (Figure 1e,f and Table S1, Supporting Information). We validated those results by genetic depletion of CHAF1A in IMR32 cells with relatively high basal CHAF1A levels (Figures S1 and S2i, Supporting Information). The conditional knockdown (KD) of CHAF1A induced cell cycle arrest in G0/G1 phase (*p* < 0.01), suppression of the G1/S checkpoint proteins CDK2 and CDK4, and stabilization of p21 and p27, the respective inhibitors of CDK2 and CDK4 (Figure S2i, Supporting Information). These results are consistent with our in vitro gain‐of‐function studies and suggest that CHAF1A plays a central role in promoting cell cycle progression and proliferation. To determine whether CHAF1A has oncogenic capacity in vitro, we generated NIH‐3T3 fibroblasts with inducible CHAF1A expression (3T3‐CHAF1A) in the presence or absence of constitutive oncogenic HRAS co‐expression (Figure S2j, Supporting Information). Both CHAF1A and HRAS expression separately induced colony formation in the NIH‐3T3 fibroblasts (*p* < 0.05 and *p* < 0.0001, respectively; Figure S2j, Supporting Information). Notably, CHAF1A together with HRAS expression resulted in significantly more colonies than HRAS expression alone (*p* < 0.001), suggesting that high CHAF1A cooperates with oncogenic HRAS to promote oncogenesis. To further determine the contribution of CHAF1A to NB oncogenesis, we orthotopically implanted SHEP cells with and without conditional CHAF1A overexpression (Figure 1a) into the renal capsule of nude mice and then assessed tumor growth. SHEP cells exhibit very poor engraftment under basal conditions. Mice were fed with control chow (CHAF1A OFF) or doxycycline (DOX)‐containing chow to induce CHAF1A expression (CHAF1A ON), and tumor formation was assessed 5 weeks after xenograft implantation (Figure 1g, left). We found that turning on CHAF1A significantly increases the engraftment rate and the average tumor weight (*p* = 0.012; CHAF1A OFF: engraftment rate 22%, tumor weight 0.15 ± 0.07 g; CHAF1A ON: engraftment rate 75%, tumor weight 0.54 ± 0.24 g; Figure 1g, right). These results suggest that activation of CHAF1A expression promotes NB cell malignancy and oncogenesis.

### 2.2 CHAF1A Blocks Neuronal Differentiation

**Figure 2:**
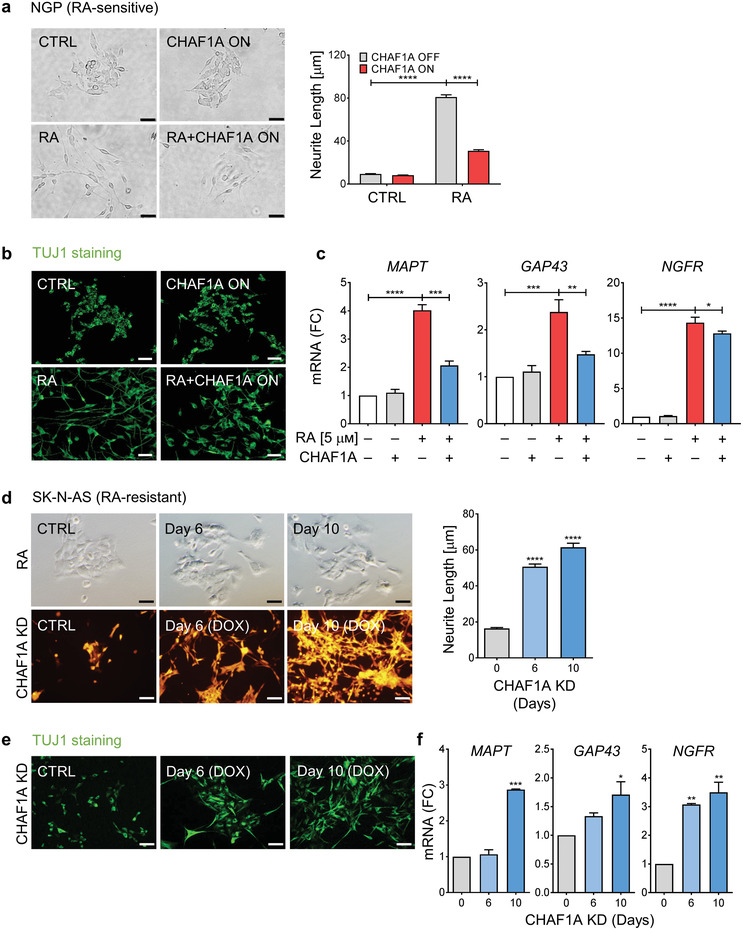
CHAF1A blocks RA‐induced cell differentiation. a) Bright field images of neurite outgrowth and quantification of neurite length. RA‐sensitive NGP cells were treated with RA (5 *μ*м) in the presence or absence of CHAF1A induction for 72 h. Neurite length was quantified by Image J2 and presented as mean ± SEM (*n* > 300, two biological replicates); ****p < 0.0001; two‐way ANOVA with Tukey's multiple comparisons test. Scale bar = 50 µm. b) TUJ1 immunofluorescence staining. Scale bar = 50 µm. c) qPCR analysis of neuron‐specific marker genes (*MAPT*, *GAP43*, and *NGFR*). Mean ± SD (*n* = 3); *p < 0.05, **p < 0.01, ***p < 0.001, ****p < 0.0001; two‐sided unpaired *t*‐test. d) RA treatment (10 *μ*м) and CHAF1A conditional KD (0–10 days) in RA‐resistant SK‐N‐AS cells. Neurite length is quantified by Image J2 and presented as mean ± SEM (*n* > 150, two biological replicates); ****p < 0.0001; one‐way ANOVA with Dunnett's multiple comparisons test. Scale bar = 100 µm. e) TUJ1 immunofluorescence staining. Scale bar = 50 µm. f) qPCR analysis of neuron‐specific genes (*MAPT*, *GAP43*, and *NGFR*). Mean ± SD (*n* = 2); *p < 0.05, **p < 0.01, ***p < 0.001; one‐way ANOVA with Dunnett's multiple comparisons test. KD = knockdown. FC = fold change.

The differentiation inducer 13‐*cis*‐RA is commonly used as maintenance therapy for high‐risk patients with NB.^[^ ^9^ ^]^ We previously showed that RA downregulates CHAF1A expression, and CHAF1A depletion promotes neuronal differentiation in RA‐sensitive NB cells.^[^ ^26^ ^]^ To investigate the role of CHAF1A in RA‐induced neuronal differentiation, we generated NGP‐CHAF1A cells where CHAF1A expression can be conditionally turned on (Figure S2e, Supporting Information). In response to RA, MNA NGP cells cease proliferation and exhibit neurite outgrowth, a morphologic feature of differentiation. Conditional CHAF1A overexpression in NGP cells restricted RA‐induced neurite outgrowth, as determined by quantification of neurite length (*p* < 0.0001, **Figure** **2a**) and immunofluorescence staining of the neuron‐specific class III *β*‐tubulin (TUJ1) (Figure 2b), a marker of mature neurons. In addition, CHAF1A overexpression in NGP cells attenuated the RA‐mediated upregulation of well‐characterized markers of terminal neuronal differentiation (*MAPT* *, GAP43*, and *NGFR*) (*p* < 0.05, Figure 2c). Similarly, CHAF1A overexpression blocked RA‐induced neuronal outgrowth and suppressed neuronal gene expression in a second MNA cell line (LAN5, Figure S3a–c, Supporting Information), as well as two non MNA cell lines (SK‐N‐SH and CHLA255, Figure S3d–i, Supporting Information). Collectively, our results indicate that CHAF1A opposes RA‐induced cell differentiation independently of MYCN amplification status.

Due to disease heterogeneity, patients with NB exhibit limited and varied responses to RA therapy. Previous studies have shown that monoallelic deletion of the retinoid metabolism gene *DHRS3*, loss of the tumor suppressor *NF1*, and suppression of *ZNF423*, a transcriptional coactivator of the RA receptors, resulted in resistance to RA‐induced differentiation.^[^ ^28^, ^29^, ^30^ ^]^ Here, to determine whether CHAF1A contributes to RA resistance in NB, we conditionally knocked down CHAF1A in RA‐resistant SK‐N‐AS cells^[^ ^31^ ^]^ (Figure S3j, Supporting Information). Wild‐type SK‐N‐AS cells fail to differentiate into neuron‐like cells in response to RA. However, the depletion of CHAF1A in SK‐N‐AS cells inhibited proliferation (Figure S3k, Supporting Information) and induced profound neurite outgrowth after a latency of 6 to 10 days (*p* < 0.0001, Figure 2d). The morphological differentiation induced by CHAF1A KD was validated by TUJ1 immunofluorescence staining (Figure 2e), and the molecular differentiation was confirmed by the upregulation of the neuronal markers *MAPT*, *GAP43*, and *NGFR* (*p* < 0.05, Figure 2f). These results indicate that depletion of CHAF1A is sufficient to promote neuronal differentiation in NB cells that fail to respond to RA.

High‐risk NBs are thought to arise from a small number of recurrent genetic alterations that block the ability of NCCs to differentiate.^[^ ^32^ ^]^ To assess the contribution of CHAF1A to the block of NCC differentiation, we employed zebrafish embryos as a model. We first investigated whether CHAF1A is expressed during NCC differentiation in vivo. In zebrafish, NCCs are specified between 11 hours post fertilization (hpf) and 20 hpf, become migratory soon thereafter, and begin the first phase of differentiating into derivatives between 24 hpf and 36 hpf (**Figure** **3a**). At 16 hpf, we detected the expression of *chaf1a* together with the early NC marker *sox9b* and the pan NC marker *crestin* within the dorsal neuroepithelium of the zebrafish embryos, along with the expression of *chaf1a* and *crestin* within delaminating NCCs along the cranial region (Figure 3b), indicating that *chaf1a* is expressed in specified and delaminating NCCs in early embryos. By 24 hpf, *chaf1a* was maintained in the dorsal neural tube along with *sox9b* and *crestin* (Figure 3b), indicating that *chaf1a* continues to be expressed in undifferentiated NCCs. Notably, *chaf1a* and *mycn* were co‐expressed (Figure 3c, arrow heads) in the cranial and vagal NCC regions of the embryo at 16 hpf and 24 hpf. Because MYCN expression fades during differentiation of NCCs into ganglia,^[^ ^33^, ^34^ ^]^ we assessed both *chaf1a* and *mycn* expression within late developing NCCs and their derivatives using a single‐cell zebrafish atlas,^[^ ^35^ ^]^ which includes thousands of isolated NCCs and NCC‐derived cell populations (*sox10*+) captured at two stages during embryonic to larval transition (48–50 hpf and 68–70 hpf). We found that both *chaf1a* and *mycn* were co‐expressed in non‐neuronal NCC derivatives at 48–50 hpf (Figure 3d); however, their levels fade in neuronal NCC derivatives (Figure 3d, upper panel tan dotted line). To analyze *chaf1a* expression during neuronal fate acquisition, we compared the expression of *chaf1a* with that of *elavl3*, a marker for neuronal differentiation, at 48–50 hpf (Figure 3e, upper panel) and 68–70 hpf (Figure 3e, bottom panel). *chaf1a* is primarily expressed in non‐neuronal NCC derivatives; in contrast, cells with neuronal fate (*elavl3*+) do not express *chaf1a* (Figure 3e, upper panels tan dotted lines). Furthermore, at 48 hpf, neurons differentiating into cranial ganglia (Figure 3e, lower panel white arrowheads) express *elavl3* but do not express *chaf1a*, while the surrounding non‐neuronal tissue (Figure 3e, lower panel red arrowheads) expresses *chaf1a* but not *elavl3*. At 70 hpf, the developing cranial ganglia continue to express *elavl3*; however, both *chaf1a* and *mycn* expression almost completely disappear, even in the surrounding tissue (Figure 3e,f). These data indicate that *chaf1a* is co‐expressed with *mycn* in undifferentiated NCC, but not in differentiated ganglia, suggesting that depletion of CHAF1A and MYCN are required for NCC to differentiate towards a neuronal lineage in vivo.

Figure 3CHAF1A blocks NC differentiation. a) Schematic presentation of early NCC events during zebrafish development. hpf = hours post fertilization. b) Spatial‐temporal expression of *sox9b*, *crestin*, and *chaf1a* in 16 hpf and 24 hpf embryos by hybridization chain reaction (HCR). A (anterior), P (posterior), D (dorsal), and V (ventral) axes shown in upper left corner. c) Spatial‐temporal expression of *chaf1a* and *mycn* in 16 hpf and 24 hpf embryos by HCR. d) Top: tSNE plots with relative expression levels of *chaf1a* and *mycn* in NCC and NCC derivatives at 48–50 hpf (red = *chaf1a*, blue = *mycn*, magenta = *both*). Bottom: HCR against *chaf1a* and *mycn* in 48 hpf embryos. Arrowheads: populations co‐expressing *chaf1a* and *mycn*. e) Top: tSNE plots with relative expression of *chaf1a* and *elavl3* in NCC and NCC‐derivatives at 48–50 hpf and at 68–70 hpf (red = *chaf1a*, blue = *elavl3*, magenta = *both*). Bottom: HCR against *chaf1a* and *elavl3* in 48 hpf and 70 hpf embryos. White arrowheads: developing cranial ganglia (*elavl3*+); red arrowheads: surrounding non‐neuronal tissue (*chaf1a*+). f) Top: tSNE plots with relative expression of *mycn* and *elavl3* in NCC and NCC‐derivatives at 68–70 hpf (red = *mycn*, blue = *elavl3*, magenta = *both*). Bottom: HCR against *mycn* and *elavl3* in 70 hpf embryos. White arrowheads: developing cranial ganglia (*elavl3*+). g) Diagram for ectopic expression of human CHAF1A in zebrafish NCCs. h) Percentage of GFP+/mCherry+ or GFP+/CHAF1A+ clones that also express Elavl3. i) Representative image from a *sox10*: mCherry‐IRES‐EGFP and a *sox10*:CHAF1A‐IRES‐EGFP injected embryo. Markers: EGFP (green), gene of interest (GOI) either mCherry or CHAF1A (red), and Elavl3 (cyan). White arrowheads: GFP+/GOI+, GFP+/mCherry+, or GFP+/CHAF1A+ clones; tan arrowheads: GFP+/GOI+/Elavl3+ clones. j) CHAF1A expression in neurons versus NCCs in a hESCs‐derived NCC induction and differentiation model. Left: schematic representation of the hNC model. Middle: TFAP2A and TUJ1 immunofluorescence staining in NCCs and neurons, respectively. Right: qPCR analysis of NCC markers (*SOX9* and *TFAP2A*) and neuron markers (*TUBB3*, which encodes TUJ1, and *MAPT*) in NCCs and neurons. Data are presented as the mean ± SD (*n* = 2); two‐sided unpaired *t*‐test, *p < 0.05, **p < 0.01. k) Left: schematic representation of NCC induction and RA‐induced differentiation into mature neurons with or without CHAF1A overexpression. Middle: immunofluorescence staining of TUJ1 with or without CHAF1A overexpression. Right: percentages of TUJ1 positive cells are quantified with Image J2. Mean ± SD (*n* = 6); two‐sided unpaired *t*‐test, ****p < 0.0001. Scale bars = 100 µm in (a–f), (j), (k), and uncropped images in (i); Scale bars = 25 µm for cropped images in (i). y = yolk sac, e = developing eye, b = developing brain, sc = developing spinal cord.

**Figure:**
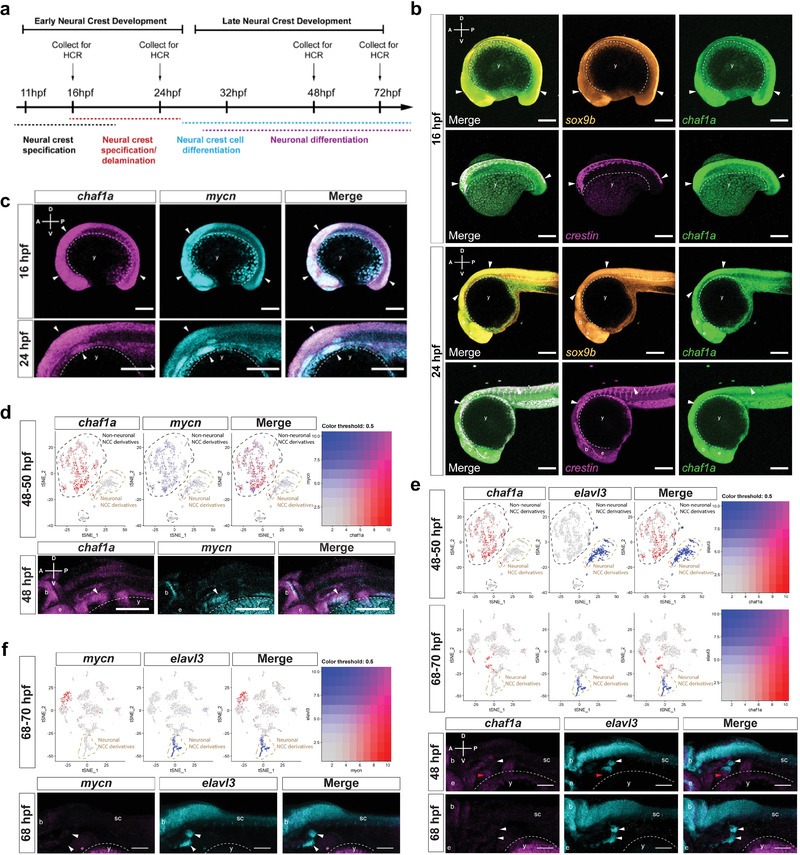

**Figure:**
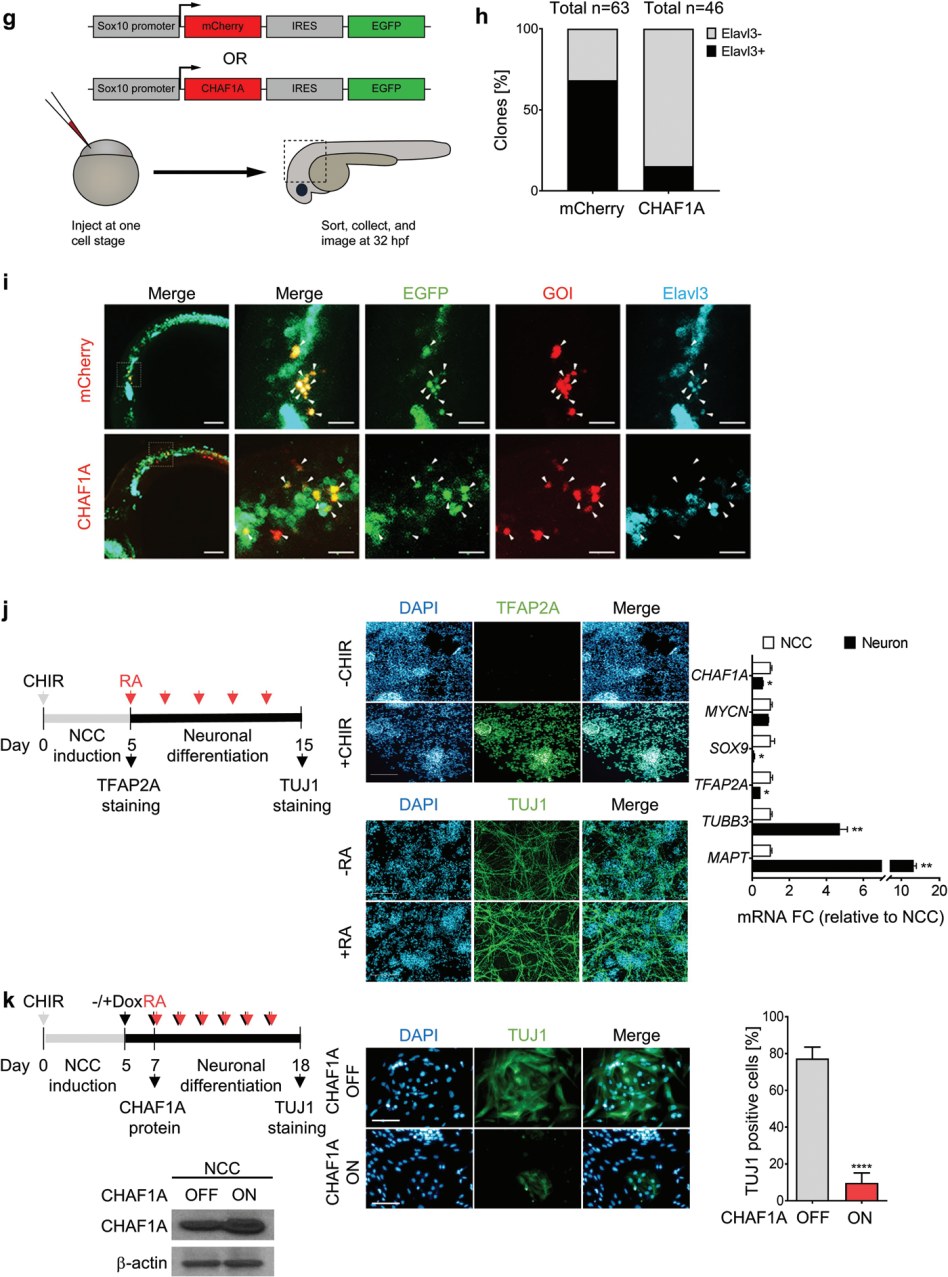

To then determine whether CHAF1A expression within the NCC lineage is sufficient to block NCC differentiation into neurons in vivo, we injected ectopic expression constructs containing human CHAF1A or mCherry as a control, into zebrafish embryos at the 1‐cell stage. Gene expression was controlled by a *sox10* promoter,^[^ ^36^ ^]^ which drives expression in zebrafish NCCs during early NCC development (Figure 3g). At 32 hpf, 68% of the embryonic cells that expressed mCherry co‐expressed the neuronal differentiation marker Elavl3, whereas only 15% of the cells that ectopically expressed CHAF1A co‐expressed Elavl3 (Figure 3h,i). However, this attenuation of differentiation was not associated with a significant compensatory increase of undifferentiated precursors, as no significant changes in proliferation (determined as percentage of phospho‐Histone H3 (pHH3) positive cells at 32 hpf) were detected between CHAF1A‐expressing and mCherry‐expressing control cells (*p* = 0.72, Figure S4a, Supporting Information). These results indicate that CHAF1A expression in NCCs is sufficient to reduce NCC differentiation into neurons.

To validate the role of CHAF1A in blocking NC differentiation, in parallel we employed an in vitro developmental model in which human embryonic stem cells (hESCs) differentiate into NCCs upon activation of Wnt signaling. The NCCs then differentiate further into neurons in response to RA treatment.^[^ ^37^ ^]^ Treatment with a Wnt activator (CHIR 99 021) efficiently induced hESCs differentiation into NCCs, as evidenced by the upregulation of the canonical NCC marker TFAP2A (Figure 3j). Subsequent treatment with RA caused the NCCs to differentiate further into dopaminergic neurons after a latency of about 10 days, as evidenced by axon maturation and expression of the canonical neuronal marker TUJ1 (Figure 3j). We found that *CHAF1A* expression was significantly downregulated in the differentiated neurons compared with that in the precursor NCCs (Figure 3j), confirming that normal *CHAF1A* expression is restricted to undifferentiated cells. By comparison, *MYCN* expression did not change. This suggests that most likely MYCN is not required for differentiation of dopaminergic neurons from NCCs in our hESC model (Figure 3j) and supports an early role for MYCN within the neural tube prior to NC specification.^[^ ^33^ ^]^ To determine whether CHAF1A could impair RA‐induced NCC differentiation into neurons, we ectopically expressed CHAF1A in NCCs using a Tet‐ON conditional system (Figure 3k). We then treated the NCCs with RA in the presence or absence of conditional CHAF1A overexpression (Figure 3k). RA effectively induced neuronal differentiation in NCCs, as evidenced by TUJ1/DAPI immunofluorescence staining and quantification of TUJ1‐positive cells. However, in the presence of CHAF1A overexpression, RA treatment failed to induce terminal differentiation of the NCCs into neurons (*p* < 0.0001, Figure 3k and Figure S4b, Supporting Information). These results indicate that CHAF1A expression is sufficient to block NCC differentiation. Altogether, our in vivo and in vitro results suggest that CHAF1A blocks NC differentiation during development.

### 2.3 CHAF1A Alters Neuronal Differentiation Programs and Rewires NB Metabolism

**Figure 4:**
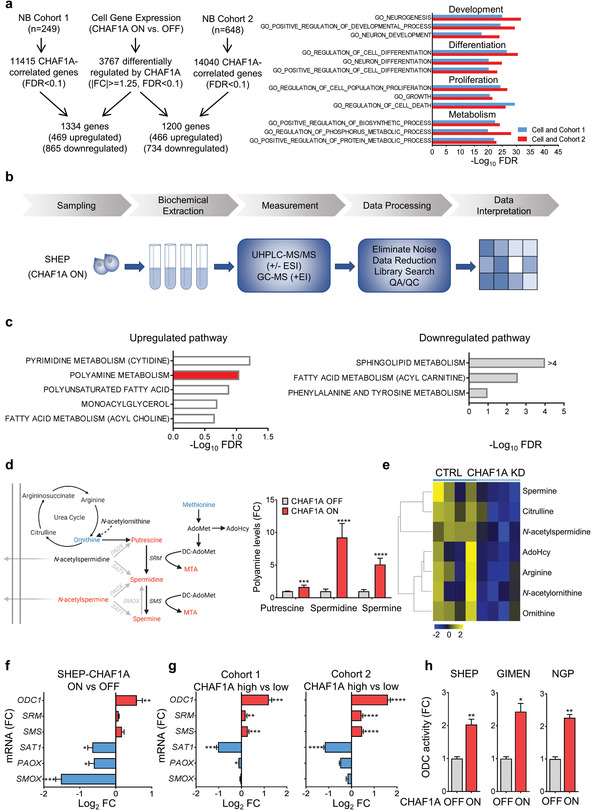
CHAF1A gene expression and pathway analyses of NB cells and patients. a) Left: overlap of differentially expressed genes (DEGs, |(fc)| > = 1.25, FDR < 0.1) between control (CHAF1A OFF) and CHAF1A‐overexpressing SHEP cells (CHAF1A ON, 96 h) and *CHAF1A*‐correlated genes (FDR < 0.1) in patient cohort 1 (*n* = 249) and 2 (*n* = 648). Right: GO pathway enrichment analysis of the overlapped genes (ranked by −Log_10_FDR, FDR<0.05). b) Work flow of the metabolomics analysis: global metabolomics analysis was performed by GC‐MS and LC‐MS (DiscoveryHD4 platform, Metabolon Inc.) in CHAF1A‐overexpressing SHEP cells (DOX 1 µg mL^−1^ for 0, 24, and 72 h, *n* = 5). c) Metabolite enrichment analysis depicts the pathways significantly up‐ and down‐regulated by CHAF1A (DOX 24 h, FDR < 0.25); Benjamini–Hochberg corrected two‐sided homoscedastic *t*‐test. d) Left: schematic presentation (redrawn from Gamble et al.^[^ ^52^ ^]^) of the polyamine pathway with metabolite changes in SHEP cells with or without CHAF1A overexpression for 24 h (red = upregulated metabolites, *p* ≤ 0.05; blue = downregulated metabolites, *p* ≤ 0.05). Right: polyamine levels in SHEP cells with or without CHAF1A overexpression for 24 h. Data are mean ± SD (*n* = 5). e) Targeted polyamine analysis in IMR32 cells with conditional KD of CHAF1A (DOX 1 µg mL^−1^ for 5 days). Differential metabolites (FDR < 0.25) are presented in the heatmap (yellow = upregulated; blue = downregulated) (*n* = 4). f) Polyamine synthetic and catabolic gene expression in SHEP cells with or without CHAF1A overexpression (24 h). Data are mean ± SD (*n* = 2); *p < 0.05, **p < 0.01, ***p < 0.001; two‐sided unpaired *t*‐test. g) Polyamine gene expression in patients with high and low *CHAF1A* expression (average CHAF1A mRNA expression ± 1SD, Figure 1) in patient cohorts 1 and 2. Data are mean ± SEM (*n* = 44 in cohort 1 and *n* = 107 in cohort 2); *p < 0.05, **p < 0.01, ***p < 0.001, ****p < 0.0001; two‐sided unpaired *t*‐test. h) ODC1 activity in SHEP, GIMEN, and NGP cells with or without CHAF1A overexpression (8 h). One unit is defined as the fluorescence change per minute. Data are normalized by the protein amount and presented as the fold change compared to control (mean ± SD, *n* = 2); *p < 0.05, **p < 0.01; two‐sided unpaired *t*‐test. MTA = 5'‐methylthioadenosine; AdoMet = *S*‐(5'‐Adenosyl)‐*L*‐methionine; AdoHyc = *S*‐(5′‐Adenosyl)‐*L*‐homocysteine; FC = fold change.

To investigate the molecular mechanisms by which CHAF1A promotes oncogenesis, we performed gene expression profiling of SHEP cells with conditional CHAF1A overexpression at 0, 24, 72, and 96 h after CHAF1A induction (Table S2, Supporting Information). A total of 416 genes were differentially expressed between control cells and CHAF1A‐overexpressing cells at 96 h after CHAF1A induction (absolute fold change ≥ 2; FDR < 0.1; 143 upregulated and 273 downregulated; Figure S5a, Supporting Information). GO pathway enrichment analysis indicated that the differentially expressed genes were enriched in pathways associated with development, differentiation, proliferation, and metabolism (FDR ≤ 0.05; Figure S5a, Supporting Information). To select genes relevant to primary NB tumors, we overlapped the differentially expressed genes in SHEP cells (absolute fold change ≥ 1.25; FDR < 0.1) with CHAF1A‐correlated genes (FDR < 0.1) in two NB patient cohorts (cohort 1: TARGET, *n* = 249; cohort 2: GSE45547, *n* = 648). A total of 1334 genes regulated by CHAF1A in SHEP cells correlated with CHAF1A expression in cohort 1 (469 upregulated in cells and positively correlated with CHAF1A; 865 downregulated in cells and negatively correlated with CHAF1A, **Figure** **4a**, left). Similarly, 1200 genes regulated by CHAF1A in SHEP cells correlated with CHAF1A expression in cohort 2 (466 upregulated, 734 downregulated, Figure 4a, left). The top enriched GO pathways included development, differentiation, proliferation, and metabolism (FDR ≤ 0.05, Figure 4a, right). In addition, to further validate our results we overlapped the differentially expressed genes upon depletion of CHAF1A in IMR32 NB cells (GSE51978) with CHAF1A‐correlated genes in cohorts 1 and 2 (Figure S5b, left, Supporting Information). The top enriched GO categories again included development, differentiation, proliferation, and metabolism (Figure S5b, right, Supporting Information). By integrating the differentially expressed genes in SHEP cells (CHAF1A ON versus OFF) and IMR32 cells (CHAF1A KD versus CTRL) along with CHAF1A‐correlated genes in cohorts 1 and 2, we generated a list of 33 genes that are consistently regulated by CHAF1A in all systems we interrogated (Table S2, Supporting Information). We further selected a subset of genes (*n* = 10) based on their inclusion in the identified functional categories (development, differentiation, proliferation, and metabolism), and validated their expressions by qPCR in IMR32 cells upon genetic depletion of CHAF1A (Figure S5c, Supporting Information). CHAF1A downregulated *CXXC5* (CXXC finger protein 5, a retinoid‐inducible nuclear protein), *LIFR* (LIF receptor subunit alpha), both of which are positive regulators of cell differentiation,^[^ ^38^, ^39^, ^40^ ^]^ and upregulated *CRABP1* (cellular retinoic acid binding protein 1), which blocks cell differentiation in NB.^[^ ^41^ ^]^ In addition, CHAF1A downregulated *SLC41A2* (solute carrier family 41 member 2) and *NR3C1* (nuclear receptor subfamily 3 group C member 1, glucocorticoid receptor), which play a role in metabolic homeostasis.^[^ ^42^, ^43^ ^]^ Taken together, those results suggest that CHAF1A overexpression culminates in transcriptional alterations that lead to restriction of differentiation and metabolic reprogramming.

These expression analyses, together with our previous findings,^[^ ^26^ ^]^ suggest that CHAF1A is involved in the reprogramming of cell metabolism. This function has not been reported. To investigate the metabolic changes induced by CHAF1A, we performed untargeted metabolomics analyses of SHEP cells upon conditional overexpression of CHAF1A using the DiscoveryHD4 platform (Metabolon Inc.) (Figure 4b). A total of 293 metabolites were significantly altered (134 upregulated and 159 downregulated; *p* ≤ 0.05) after CHAF1A expression was induced for 24 h (Table S3, Supporting Information). Metabolite enrichment analysis revealed that CHAF1A induction upregulates distinct pathways for pyrimidine, polyamine, and polyunsaturated fatty acid metabolism (FDR < 0.25), whereas it downregulates pathways for sphingolipid, acyl carnitine‐related fatty acid, and phenylalanine and tyrosine metabolism (FDR < 0.25, Figure 4c and Table S3, Supporting Information). Notably, the polyamine pathway was consistently enriched both after 24 and 72 h of CHAF1A induction (FDR < 0.25, Figure 4c, and Figure S5d and Table S3, Supporting Information). Polyamine homeostasis depends on the biosynthesis, catabolism, and transport of three major polyamines: spermidine, spermine, and putrescine. Polyamines are known for promoting protein synthesis and cell proliferation,^[^ ^44^ ^]^ especially in the context of MYCN amplification.^[^ ^45^ ^]^ By employing mass spectrometry, we showed that CHAF1A induction in SHEP cells promotes the accumulation of spermidine and spermine (by 9.2 fold and 5.1 fold, respectively; *p* < 0.0001) and also 5′‐methylthioadenosine (MTA; *p* < 0.01), the intermediate metabolite of spermidine and spermine synthesis (Figure 4d and Table S3, Supporting Information). Correspondingly, the levels of the polyamine‐precursor metabolites ornithine and methionine were reduced (*p* < 0.0001), while that of the catabolic form of spermine (*N*‐acetylspermine) was increased (*p* < 0.0001; Figure 4d and Table S3, Supporting Information), suggesting that CHAF1A promotes polyamine accumulation. To confirm the role of CHAF1A in polyamine metabolism, we performed targeted polyamine analysis by liquid chromatography‐mass spectrometry in IMR32 cells where CHAF1A is conditionally silenced (DOX 1 µg mL^−1^ for 5 days, Figure 4e and Table S4, Supporting Information). Spermine and polyamine precursors (ornithine, citrulline, and arginine) were all significantly downregulated upon CHAF1A KD (FDR < 0.25), supporting the notion that CHAF1A activates polyamine synthesis. We then determined the effect of CHAF1A on gene expression in the polyamine metabolic pathway. Activation of CHAF1A induces the expression of genes for polyamine synthesis (*ODC1*, *SRM*, and *SMS*) and suppresses the expression of genes for polyamine catabolism (*SAT1*, *PAOX*, and *SMOX*)^[^ ^44^ ^]^ (*p* < 0.05; Figure 4f). In addition, in both patient cohorts 1 and 2, the mRNA levels of polyamine metabolism genes significantly correlate with CHAF1A expression levels (*p* < 0.05; Figure 4g) and clinical outcome (*p* < 0.01^[^ ^46^ ^]^). Those results suggest that CHAF1A promotes polyamine accumulation by upregulating polyamine synthetic pathways and downregulating polyamine catabolic pathways. Ornithine decarboxylase (ODC1) is the rate‐limiting enzyme of polyamine synthesis.^[^ ^44^ ^]^ CHAF1A expression in NB cells not only promotes *ODC1* transcription but also significantly enhances ODC1 activity (*p* < 0.05; Figure 4h), suggesting that CHAF1A activates polyamine biosynthesis to sustain cell growth.

### 2.4 Targeting Polyamine Metabolism Restores Neuronal Differentiation and Blocks CHAF1A's Oncogenic Functions

To determine whether polyamines contribute to CHAF1A‐mediated suppression of differentiation and oncogenesis, we tested the ability of DFMO, an irreversible inhibitor of ODC1,^[^ ^47^, ^48^ ^]^ to oppose the effects of CHAF1A on RA‐induced differentiation and cell cycle progression. CHAF1A consistently blocks RA‐induced differentiation in NGP‐CHAF1A cells (*p* < 0.0001; **Figure** **5a**,b, pink arrows), whereas treatment with DFMO almost completely reverses this effect and restores RA sensitivity, as evidenced by neurite length quantification (*p* < 0.0001; Figure 5a, red arrows) and TUJ1 staining (Figure 5b, red arrows). Moreover, DFMO completely blocks CHAF1A‐induced cell cycle progression (*p* < 0.05; Figure 5c). Those results suggest that CHAF1A blocks differentiation and promotes cell cycle progression in part by upregulating polyamine synthesis.

Figure 5Inhibition of polyamine synthesis restores neuronal differentiation. a,b) Neurite length and TUJ1 immunofluorescence staining in NGP‐CHAF1A cells treated with RA (5 *μ*м), DOX (1 µg mL^−1^), and DFMO (0.5 mм) for 72 h. Data are mean ± SEM (*n* > 300); ****p < 0.0001; one‐way ANOVA with Tukey's multiple comparisons test. Scale bar = 50 µm. c) Cell cycle analysis of NGP‐CHAF1A cells treated with DOX (1 µg mL^−1^) or DFMO (0.5 mм) for 72 h. Data are mean ± SD (*n* = 2); *p < 0.05, **p < 0.01, ***p < 0.001, ****p < 0.0001; two‐way ANOVA with Tukey's multiple comparisons test. d) Neuronal outgrowth of IMR32 CHAF1A KD cells in the presence or absence of ODC1 overexpression. Neurite length is quantified using Image J2 and presented as mean ± SEM (*n* > 300); ****p < 0.0001; one‐way ANOVA with Tukey's multiple comparisons test. Scale bar = 50 µm. e) Cell cycle analysis of IMR32 CHAF1A KD cells in the presence or absence of ODC1 overexpression. Data are mean ± SD (*n* = 2); ****p < 0.0001; two‐way ANOVA with Tukey's multiple comparisons test. f) Cell viability of LAN5, IMR32, CHLA255, and SK‐N‐AS cells treated with increasing concentrations of DFMO single agent, RA single agent, and their combination (combo). Cell viability of LAN5 shCTRL and shCHAF1A cells treated with increasing doses of RA. Data are mean ± SD (*n* = 3). †synergy with CI < 1; **p < 0.01; ***p < 0.001; two‐sided unpaired *t*‐test. g) Apoptosis of LAN5, IMR32, CHLA255, and SK‐N‐AS cells treated with DFMO, RA, and combo (IC_50_–_75_). Apoptosis of LAN5 shCTRL and shCHAF1A cells treated with RA (IC_50_). Data are mean ± SD (*n* = 3); * *p* < 0.05, ** *p* < 0.01, *** *p* < 0.001, **** *p* < 0.0001; two‐way ANOVA with Tukey's multiple comparisons test. h) Polyamine metabolites in LAN5 cells treated with DFMO (1 mм), RA (10 *μ*м), and combo for 5 days (*n* = 4). Metabolites with FDR < 0.05 in at least one comparison are shown in the heatmap (red = upregulated; blue = downregulated); two‐way ANOVA with original FDR method of Benjamini and Hochberg. The relative abundance of putrescine and spermidine are presented in box and whiskers plots. # indicates FDR < 0.05. i) Top, scheme of RA+DFMO study in LAN5 luc orthotopic xenograft model. Mice were treated with vehicle (1% methylcellulose, p.o., b.i.d., 5 days per week), RA (p.o., 40 mg kg^−1^ b.i.d., 5 days per week), DFMO (2% in sterile water, replaced weekly), and their combination for three weeks. Bottom left, tumor weights post treatment. Mean ± SEM (*n* = 8–11); Mann–Whitney test. Bottom right, cleaved caspase‐3 staining and quantification in tumors. Scale bar = 20 µm. Mean ± SEM (*n* = 6); Mann–Whitney test. j) Top, scheme of RA study in LAN5 luc shCHAF1A versus shCTRL orthotopic xenograft model. Mice were treated with vehicle (1% methylcellulose, p.o., b.i.d., 5 days per week) or RA (p.o., 40 mg kg^−1^ b.i.d., 5 days per week) for 3 weeks. Bottom left, tumor weights post treatment. Mean ± SEM (*n* = 9–10); Mann–Whitney test. Bottom right, cleaved caspase‐3 staining and quantification in tumors. Scale bar = 20 µm. Mean ± SEM (*n* = 5–6); Mann–Whitney test. FC = fold change; ns = not significant.

**Figure:**
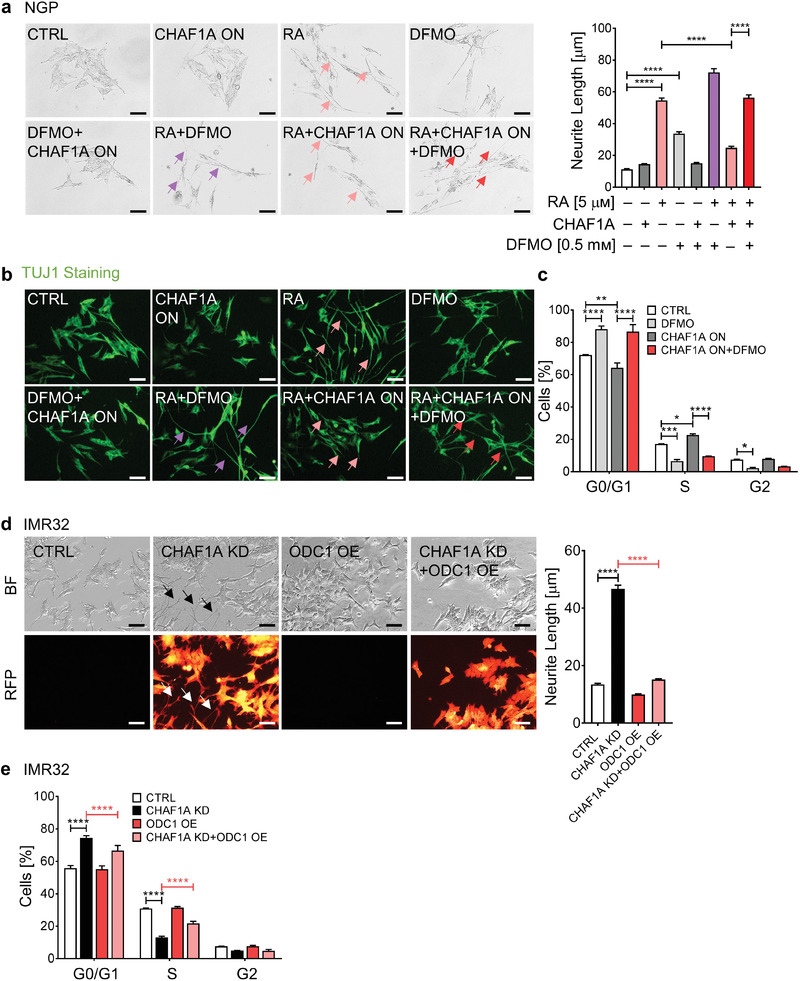

**Figure:**
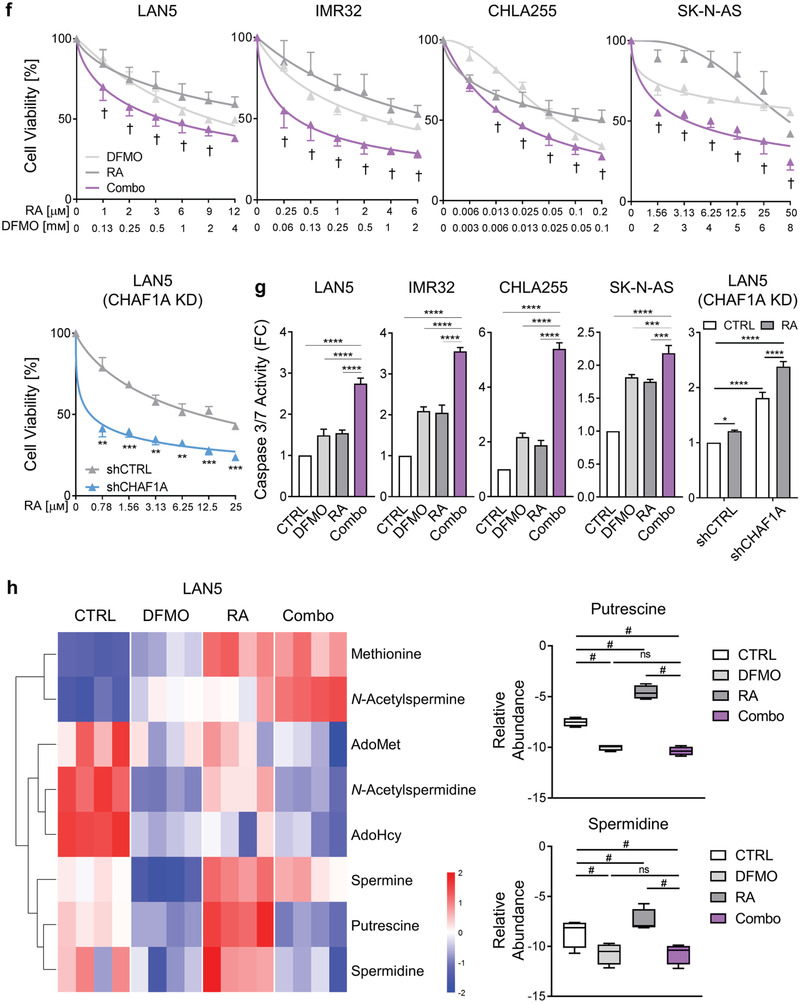

**Figure:**
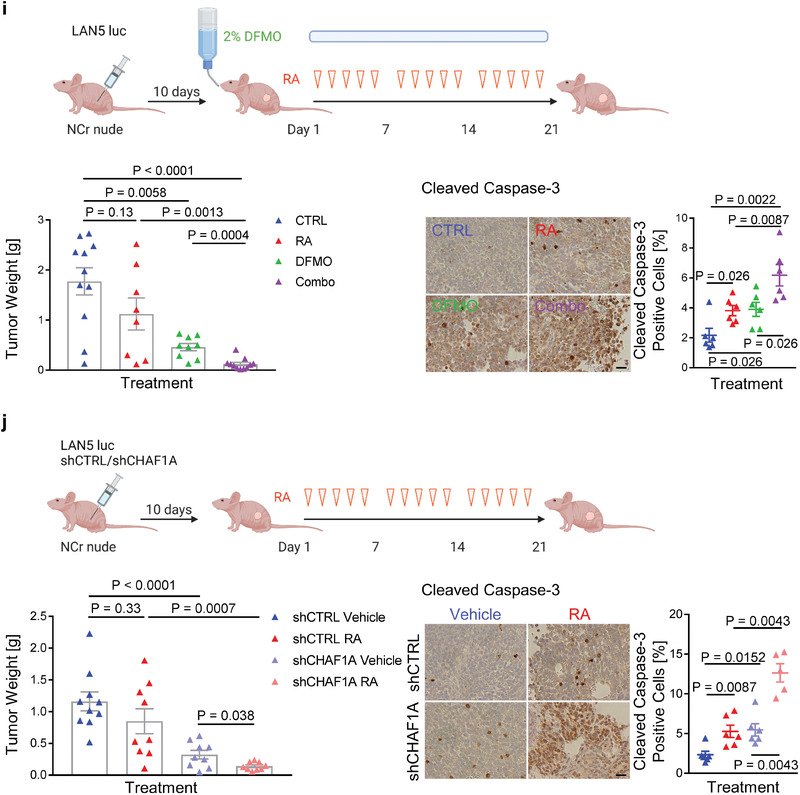

To determine whether genetic activation of polyamine metabolism could reverse the cellular phenotype induced by CHAF1A depletion, we overexpressed ODC1 in IMR32 cells in the presence or absence of inducible anti‐CHAF1A shRNA (Figure S6a, Supporting Information). Genetic KD of CHAF1A induces cell differentiation as evidenced by neurite outgrowth (Figure 5d); however, ectopic overexpression ODC1 reverses this effect, suggesting that activation of polyamine synthesis contributes to CHAF1A‐mediated suppression of differentiation (Figure 5d). In addition, genetic KD of CHAF1A consistently induces cell cycle arrest in G0/G1 phase (Figure 5e); however, ectopic overexpression of ODC1 also reverses also this effect, promoting cell cycle progression from G0/G1 phase to S phase (Figure 5e).

Because our results suggest that CHAF1A opposes RA‐induced differentiation in part by polyamine accumulation, we asked whether inhibition of polyamine metabolism could enhance the clinical efficacy of RA therapy. DFMO enhances the cell differentiation induced by RA in NGP cells (Figure 5a,b, purple arrows) and two additional RA‐sensitive cell lines (LAN5, MNA, and CHLA255, non MNA) as evidenced by neurite outgrowth (*p* < 0.0001) and TUJ1 staining (Figure S6b–e, Supporting Information). DFMO also improves RA‐mediated inhibition of cell viability (Figure 5f and Figure S6f, Supporting Information, † indicates synergistic effect of DFMO+RA with CI < 1) and cell cycle progression (Figure S6g, Supporting Information) in a panel of NB cells (MNA: LAN5, IMR32, Kelly; non MNA: CHLA255, SH‐SY5Y, SK‐N‐AS). Moreover, the combination therapy (DFMO+RA) induces higher cell apoptosis compared to single agents (*p* < 0.01, Figure 5g). Importantly, DFMO+RA phenocopies the effects of CHAF1A depletion+RA (Figure 5f,g), suggesting that targeting polyamine synthesis is a promising approach to enhance the effectiveness of differentiation‐based therapies (e.g., RA). Because RA is known to increase putrescine levels,^[^ ^49^ ^]^ we sought to determine whether activation of polyamine metabolism by RA plays a role in the anti‐tumor activity of DFMO+RA. Thus, we performed targeted polyamine analysis via liquid chromatography‐mass spectrometry in two NB cell lines (LAN5 and IMR32) upon treatment with DFMO, RA, and their combination. Indeed, RA increased the levels of putrescine, spermidine, and other polyamine intermediates in these cells (FDR < 0.05, Figure 5h and Figure S6i and Table S4, Supporting Information). However, the combination therapy DFMO+RA completely reverted the levels of putrescine and its downstream metabolites (FDR < 0.05, Figure 5h and Figure S6i and Table S4, Supporting Information) resulting in lower cell viability (*p* < 0.001, Figure S6i, Supporting Information). These data suggest that the addition of DFMO effectively restores polyamine levels and increases the anti‐tumor activity of RA.

To validate this hypothesis, we next determined the anti‐tumor activity of the combination therapy DFMO+RA in vivo using our established orthotopic xenograft mouse model of NB.^[^ ^50^ ^]^ LAN5 cells overexpressing luciferase (LAN5 luc) were implanted into the renal capsule of 7‐week‐old female NCr nude mice. Tumor engraftment was assessed by bioluminescence imaging. Mice were then randomized and evenly allocated into four groups: CTRL (1% methylcellulose, p.o., b.i.d., 5 days per week), 13‐*cis*‐RA (p.o., 40 mg kg^−1^ b.i.d., 5 days per week), DFMO (2% in sterile water, replaced weekly), and their combination (Figure 5i). After 3 weeks of treatment, animals were euthanized and tumor weights evaluated. Single agent RA slightly reduced tumor weights without reaching significance. As expected, single agent DFMO significantly controlled tumor growth (*p* = 0.0058, Figure 5i). Notably, the combination DFMO+RA inhibited tumor growth (*p* < 0.01) and promoted apoptosis (*p* < 0.05) to a larger extent than the single therapies in this MNA model of NB (Figure 5i), without showing reduction of body mass (Figure S6j, Supporting Information). To further determine whether DFMO+RA phenocopies genetic depletion of CHAF1A+RA in terms of tumor growth inhibition, we implanted LAN5 luc shCTRL and shCHAF1A cells into the renal capsule of 7‐week‐old female NCr nude mice. Ten days after implantation, shCTRL and shCHAF1A tumor‐bearing mice were allocated into CTRL (1% methylcellulose, p.o., b.i.d., 5 days per week) or 13‐*cis*‐RA (p.o., 40 mg kg^−1^ b.i.d., 5 days per week) group for 21 days. In the absence of RA, genetic depletion of CHAF1A blocked tumor growth (*p* < 0.0001, Figure 5j), supporting our previous findings.^[^ ^26^ ^]^ Importantly, CHAF1A KD+RA phenocopied DFMO+RA and resulted again in a significant inhibition of tumor growth (*p* < 0.05) and induction of tumor apoptosis (*p* < 0.01) compared to single approach (Figure 5j). Our data suggest that targeting CHAF1A or CHAF1A‐induced polyamine metabolism effectively enhances RA activity. Because both DFMO and RA are currently used for NB therapy, our study provides the foundation for future clinical investigations testing this combination therapy.

Because CHAF1A and MYCN share biological functions such as differentiation and metabolism, we investigated the functional relationship between MYCN and CHAF1A in NB. CHAF1A is significantly downregulated at both transcriptional and translational levels in TET‐OFF (TET‐21/N) cells when MYCN is turned off upon DOX treatment (**Figure** **6a**,b), suggesting that CHAF1A is regulated by changes in MYCN expression. To determine whether MYCN directly regulates CHAF1A, we then immunoprecipitated MYCN‐binding chromatin and performed qPCR analysis to determine the enrichment of MYCN binding at the promoter regions of *CHAF1A*. In the absence of DOX, we demonstrated a significant enrichment of MYCN binding to the *CHAF1A* promoter (canonical E‐box, Figure 6c); however, turning off MYCN fully abrogates MYCN binding, suggesting that MYCN directly targets *CHAF1A* to promote its transcription. This regulation was validated in other MNA cells (LAN5, Figure S7a, Supporting Information). On the other hand, silencing CHAF1A resulted in a time‐dependent reduction of MYCN expression both at mRNA and protein levels, along with the reduction of expression of several known MYCN targets (*NR1D1*, *PER2*, and *ODC1*) and polyamine biosynthetic genes (Figure 6d,e and Figure S7b,c, Supporting Information). Specifically, supporting our gain‐of‐function studies (Figure 4f), depletion of CHAF1A reduced polyamine biosynthesis gene expression and increased polyamine catabolism gene expression (Figure 6d and Figure S7b, Supporting Information). To determine whether depletion of CHAF1A directly affects the ability of MYCN to bind its own promoter and two MYCN‐responsive elements present in the *ODC1* promoter (*ODC1A* and *ODC1B*), we performed ChIP in IMR32 cells upon CHAF1A KD. Silencing CHAF1A abolishes MYCN binding to the tested chromatin regions (Figure S7d, Supporting Information). Because MYCN and CHAF1A positively regulate each other and c‐MYC interacts with CHAF1B,^[^ ^51^ ^]^ we hypothesized that CHAF1A and MYCN directly cooperate in the regulation of the same target gene promoters. To test this hypothesis, we performed ChIP in IMR32 cells engineered to express an inducible HA‐tag CHAF1A derivative protein. Cells were subjected to ChIP using an anti‐HA monoclonal antibody. HA‐CHAF1A was found significantly associated with the *MYCN* TSS and *ODC1* promoter regions (Figure S7e, Supporting Information). Taken together, our findings demonstrate a positive regulatory loop between MYCN and CHAF1A expression. Importantly, the two proteins bind the same *ODC1* promoter regions with increased mRNA production, suggesting a cooperative action of the two factors in the transcriptional regulation of polyamine metabolism.

Figure 6CHAF1A is a direct target of MYCN. a,b) mRNA and protein expression of CHAF1A in TET‐21/N cells when MYCN is turned off upon DOX treatment (2 µg mL^−1^, 24–96 h). GAPDH is used as housekeeping gene, CypB as protein loading control. Data are mean ± SD (*n* = 2–3); * *p* < 0.05, ** *p* < 0.01, *** *p* < 0.001, **** *p* < 0.0001; one‐way ANOVA with Dunnett's multiple comparisons test. c) MYCN ChIP‐qPCR assays in TET‐21/N cells. Input (white bars) and MYCN‐ChIP (black bars) samples were analyzed by qPCR using specific primers for *CHAF1A* (Table S5, Supporting Information). Data from two independent experiments are shown (mean ± SEM, *n* = 2). d) mRNA expression of *CHAF1A*, *MYCN*, *MYCN* targets and polyamine genes in LAN5 cells upon CHAF1A KD (DOX 1 µg mL^−1^ for 2–5 days). GAPDH served as control. Mean ± SD (*n* = 3); * *p* < 0.05, ** *p* < 0.01, *** *p* < 0.001, **** *p* < 0.0001; two‐way ANOVA with Dunnett's multiple comparisons test. e) Protein expression of CHAF1A, MYCN, and ODC1 in LAN5 cells upon CHAF1A KD (DOX 1 µg mL^−1^ for 0–10 days). CypB served as protein loading control. Mean ± SD (*n* = 2); * *p* < 0.05, ** *p* < 0.01, *** *p* < 0.001, **** *p* < 0.0001; one‐way ANOVA with Dunnett's multiple comparisons test. f) Correlation of *CHAF1A*, *MYCN*, and *MYCN* signature scores in patient cohort 1 (*n* = 249) and cohort 2 (*n* = 648). Signatures are defined in the methods section. FC = fold change.

**Figure:**
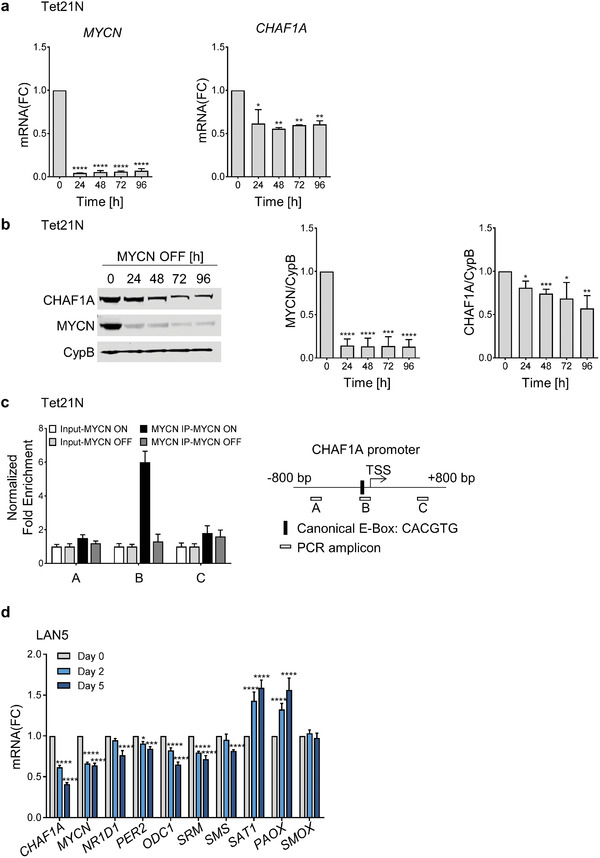

**Figure:**
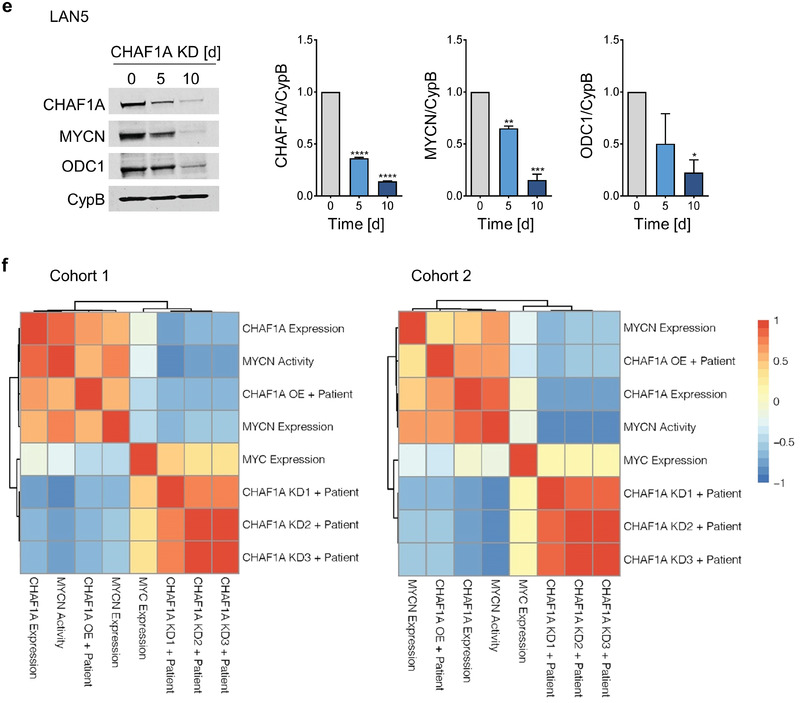

Lastly, to determine how *CHAF1A* and *MYCN* expression correlate in primary tumor samples, we first inferred several *CHAF1A* gene signatures by overlapping the differentially regulated genes in SHEP cells (CHAF1A 96 h ON versus OFF) with *CHAF1A*‐correlated genes from cohorts 1 or 2 (CHAF1A OE + patient) and by overlapping the differentially regulated genes in IMR32 cells (CHAF1A KD1 day 5, CHAF1A KD2 day 10, CHAF1A KD3 day 5 and 10 common genes) with *CHAF1A*‐correlated genes from cohorts 1 or 2 (CHAF1A KD1–3 + patient). We then analyzed the correlation of these signature activity scores and *CHAF1A* mRNA expression with *MYCN* expression/activity in both patient cohorts. Consistently, both *CHAF1A* mRNA expression and *CHAF1A* gene signature are positively correlated with *MYCN* expression and *MYCN* activity (Figure 6d and Figure S7b, Supporting Information). These data confirm a positive correlation between *CHAF1A* and *MYCN* expression/activity in large NB patient cohorts.

## 3 Discussion

Neuronal cell proliferation and differentiation are two fundamental processes that determine cell fate.^[^ ^53^ ^]^ In normal cell development, differentiation begins when cells exit the cell cycle.^[^ ^54^ ^]^ Oncogenes disrupt that process by maintaining the cell cycle and blocking differentiation, leading to oncogenesis.^[^ ^55^ ^]^ For example, the transcription factor MYCN acts as an oncogene in NB by blocking differentiation, maintaining pluripotency, and driving oncogenesis in mouse, zebrafish, and NCC‐derived models.^[^ ^5^, ^6^, ^56^ ^]^

We previously reported that high CHAF1A expression is associated with high‐risk disease and poor clinical outcome in NB.^[^ ^26^ ^]^ Here, we demonstrate that CHAF1A promotes NB cell malignancy and oncogenesis by restricting neuronal differentiation and reprogramming cell metabolism. Activation of CHAF1A expression promotes a malignant cellular phenotype; CHAF1A gain‐of‐function increases cell proliferation to a greater extent under hypoxic conditions, supporting the original identification of CHAF1A as potential HIF‐1*α* target.^[^ ^57^ ^]^ In addition, CHAF1A activates oncogenic transformation in vitro and is sufficient to initiate tumor formation in vivo, suggesting a critical role for CHAF1A in NB oncogenesis. However, further studies are needed to determine whether CHAF1A is capable of transforming NCC cells into NB in vivo. Our patient data and gain‐of‐function and loss‐of‐function studies in human NB cell lines demonstrated that CHAF1A promotes cell cycle progression and blocks RA‐induced neuronal differentiation, and that CHAF1A impairs differentiation by preventing exit from the cell cycle, thus maintaining the cells in a proliferative state. Depletion of endogenous CHAF1A promoted differentiation in both RA‐sensitive and RA‐resistant NB cells,^[^ ^26^ ^]^ suggesting an intrinsic role of CHAF1A in regulating cell differentiation. Because CHAF1A alters the expression of many RA‐responsive genes (Table S2, Supporting Information), it is possible that CHAF1A directly interacts with the RA signaling to block cell differentiation.

The mechanisms controlling NC differentiation have remained elusive. Elucidation of the molecular events governing the differentiation block in NCCs is crucial for the identification of novel targets for therapeutic intervention. Using a zebrafish embryonic model and a clonal in vivo expression system, we showed that ectopic expression of human CHAF1A in NCCs is sufficient to prevent the differentiation of those cells into neurons. Furthermore, we showed that both endogenous *chaf1a* and endogenous *mycn* are expressed in specified and delaminating NCCs but not in differentiated ganglia of zebrafish embryos during normal development, suggesting that depletion of CHAF1A and MYCN are critical events for NCC to differentiate towards a neuronal lineage in vivo. In parallel, using an in vitro human NC differentiation model, we demonstrated that overexpression of human CHAF1A is sufficient to block RA‐mediated NCC differentiation into mature neurons. Collectively, these results indicate that CHAF1A plays a critical role in determining NC cell fate and differentiation. However, the mechanisms responsible for CHAF1A's role in NCC differentiation remain unclear. A series of transcriptional and epigenetic events regulate NC development.^[^ ^2^, ^58^ ^]^ Failure in those events disrupts normal NC development and enables oncogenesis.^[^ ^59^ ^]^ CHAF1A is known to initiate a gene silencing program via DNA methylation and H3K9 trimethylation,^[^ ^19^, ^20^ ^]^ which can block NC specification and promote cell growth.^[^ ^60^, ^61^ ^]^ Further studies are needed to determine whether the epigenetic events triggered by CHAF1A enable oncogenesis.

MYCN amplification occurs in 50% of high‐risk patients and correlates with poor prognosis and treatment failure.^[^ ^62^ ^]^ Overexpression of MYCN in migratory NCCs and overexpression of c‐MYC in sympathetic neurons both drive NB oncogenesis.^[^ ^56^, ^63^ ^]^ We showed here that CHAF1A functions as a MYCN target in NB: MYCN directly binds to *CHAF1A*, and its expression correlates with *MYCN* gene signature and activity in NB patients. On the other hand, CHAF1A upregulates MYCN expression, establishing a positive regulatory loop between MYCN and CHAF1A expression and supporting a model in which CHAF1A and MYCN cooperate in the transcriptional regulation of polyamine gene expression. Although MYCN and CHAF1A present overlapping functional activities on cell cycle, differentiation and metabolism, CHAF1A may harbor unique cellular functions independently of MYCN. CHAF1A predicts patient outcome independently of MYCN status.^[^ ^26^ ^]^ Moreover, CHAF1A blocks cell differentiation independently of MYCN amplification. This may suggest that CHAF1A is epistatic to MYCN amplification. c‐MYC has been shown to interact with CHAF1B, the other component of the CAF‐1 complex, especially when c‐MYC is overexpressed.^[^ ^51^ ^]^ This suggests that high MYC(N) may affect the biological function of CAF‐1 by altering the network of inner interactions of the complex components and, as a consequence, the stoichiometry and assembly of the complex itself. Interestingly, NB cells with adrenergic phenotype (IMR32, LAN5, SK‐N‐BE, NGP, and CHLA255) appear to have higher CHAF1A expression than mesenchymal cells (SHEP and GIMEN).^[^ ^64^, ^65^ ^]^ Whether CHAF1A contributes to distinct mesenchymal versus adrenergic epigenetic landscapes in NB will need to be investigated.

Cell metabolism is constantly activated in tumors to provide biomass and energy for cell growth and proliferation.^[^ ^66^ ^]^ In addition, metabolic intermediates have emerged as key players that influence decisions of cell fate.^[^ ^66^ ^]^ We found that CHAF1A activates polyamine metabolism by inducing the expression and the activity of genes involved in polyamine synthesis, resulting in accumulation of major polyamines and sustained cell growth. Notably, polyamine metabolism is emerging as a key pathway that is activated in MYC‐driven cancers, including NB.^[^ ^45^, ^46^ ^]^ In addition, the activation of polyamine synthesis promotes the reprogramming of somatic cells into induced pluripotent stem cells, enhances the self‐renewal of mouse ESCs, and blocks the differentiation of pluripotent cells into neural precursor cells.^[^ ^67^, ^68^ ^]^ Clinical studies with DFMO have shown promising results in high‐risk NBs (NCT01059071, NCT02395666).^[^ ^48^, ^69^ ^]^ Moreover, combined inhibition of polyamine synthesis (via DFMO) and polyamine uptake (via AMXT1501) demonstrated effective anti‐tumor activity in pre‐clinical mouse models^[^ ^46^ ^]^ and will be tested in clinical trials (NCT03536728). We found that genetic and pharmacological interference with the polyamine pathway overcomes CHAF1A‐mediated suppression of differentiation and cell cycle progression, indicating that polyamines play a causative role in CHAF1A‐mediated oncogenesis. Sphingolipid metabolism was also consistently downregulated by CHAF1A activation, and further lipidomics analyses are required to assess its potential contribution to CHAF1A restriction of differentiation.

Differentiation therapy with 13‐*cis*‐RA is part of maintenance regimens for high‐risk patients with NB.^[^ ^11^, ^12^ ^]^ The efficacy of 13‐*cis*‐RA in patients with NB is limited;^[^ ^10^, ^14^ ^]^ however, we found that DFMO induces cell cycle arrest, promotes differentiation, and restores sensitivity to RA in NB cells. Our in vitro combination regimen (RA, 0.3–40 *μ*м; DFMO, 0.2–5.4 mм) is close to the plasma concentration measured in patients receiving 13‐*cis*‐RA (5–11 *μ*м)^[^ ^13^, ^70^ ^]^ and DFMO (0.05–0.17 mм).^[^ ^48^ ^]^ Importantly, the combination therapy DFMO+RA effectively blocks polyamine metabolism, exerts greater in vivo anti‐tumor activity compared to single therapies and closely recapitulates the effects of the combination CHAF1A genetic depletion+RA, suggesting that targeting CHAF1A or CHAF1A‐induced polyamine metabolism enhances RA activity. These results indicate that NB might be vulnerable to a combined approach using 13‐*cis*‐RA together with DFMO to drive NB cell differentiation and thus block tumor development. Because DFMO and RA are both currently in clinical trials and have good safety profiles, our results support their further clinical testing to improve differentiation therapy for NB.

## 4 Conclusion

NB arises from a block in NC differentiation; however, little is known about the molecular mechanisms driving the loss of differentiation in NCCs. We demonstrate, for the first time, that CHAF1A is sufficient to block neuronal differentiation in three different models (zebrafish NC, human NC, and human NB), promotes NB oncogenesis, and contributes to the resistance of NB to RA, a standard maintenance therapy. Mechanistically, we identify a novel function of CHAF1A in reprogramming polyamine metabolism to block neuronal differentiation and support oncogenesis. Inhibiting polyamine synthesis promotes NB differentiation and enhances the anti‐tumor activity of RA.

Altogether, our study provides new insights into the in vivo mechanisms contributing to NB development and progression from NC, and suggests a rapidly translatable approach to improve the clinical efficacy of differentiation‐based therapy for NB patients.

## 5 Experimental Section

### Cell Culture and Chemicals

The human NB cell lines SK‐N‐AS, IMR32, SH‐SY5Y (American Type Culture Collection), Kelly, SK‐N‐SH and SK‐N‐BE(2c) (Bernardi Lab, BCM, Houston, TX), LAN5 and CHLA255 (Metelitsa Lab, BCM, Houston, TX), SHEP and NGP (Shohet Lab, University of Massachusetts, Boston, MA) were maintained in RPMI 1640 medium (Lonza, Allendale, NJ) containing FBS (10%, Germini Bio‐Products, West Sacramento, CA), *L*‐glutamine (4 mм, Thermo Fisher Scientific, Waltham, MA), and streptomycin/penicillin (1%, Thermo Fisher Scientific). GIMEN (Altman Lab, University of Rochester, Rochester, NY) cells were maintained in DMEM medium (Thermo Fisher Scientific) containing glucose (4.5 g L^−1^), sodium pyruvate (110 mg L^−1^), FBS (10%), *L*‐glutamine (4 mм), and streptomycin/penicillin (1%). TET‐21/N cells (Perini Lab, University of Bologna, Italy) were maintained in DMEM medium (GE Healthcare Life Sciences) containing glucose (4.5 g L^−1^), FBS (10%), *L*‐glutamine (4 mм), and streptomycin/penicillin (1%). All cell lines were authenticated (STR analysis) and regularly tested for mycoplasma. Undifferentiated hESCs(H1, authenticated by WiCell, Madison, WI) were maintained on a laminin511‐coated surface in modified E8 medium supplemented with insulin (20 µg mL^−1^), human albumin (100 µg mL^−1^), holo‐transferrin (10 µg mL^−1^), TGF‐*β*1 (2 ng mL^−1^), and bFGF2 (5 ng mL^−1^). Drugs: 13‐*cis*‐RA (0.3–40 μм, Sigma, St. Louis, MO) and difluoromethylornithine (DFMO, 0.2–5.4 mм, Cayman Chemical, Ann Arbor, MI).

### Patient Cohorts and Pathway Analysis

Cohort 1 (TARGET, *n* = 249): data were profiled using the Affymetrix Human Exon Array platform and retrieved from TARGET data matrix (https://ocg.cancer.gov/programs/target/data‐matrix). Cohort 2 (Kocak, *n* = 648): data contained single‐color gene expression profiles from 649 NB tumors based on 44K oligonucleotide microarrays.^[^ ^27^ ^]^ GSM1108445 was removed from the dataset as a *CHAF1A* probe outlier. Data were downloaded from NCBI: GSE45547 (https://www.ncbi.nlm.nih.gov/geo/query/acc.cgi?acc=gse45547). High and low *CHAF1A* expression groups were defined as those individuals with *CHAF1A* expression levels one standard deviation above and below the population average, respectively. FDR for differential expression between the high and low *CHAF1A* groups were computed using a Benjamini–Hochberg corrected two‐sided homoscedastic *t*‐test. Entrez IDs for expression probes with FDR < 0.01 and absolute fold change > 2, along with the background IDs, were submitted for GSEA analysis using hallmark gene set.^[^ ^71^ ^]^

### Plasmids

To conditionally overexpress CHAF1A, a Tet‐ON inducible lentivector (pHAGE‐ind‐Lenti‐CHAF1A‐HA‐neo) was used to transduce SHEP, GIMEN, NGP, LAN5, SK‐N‐SH, CHLA255 cells, and hESCs. Lentiviruses were prepared as previously described.^[^ ^26^ ^]^ In the zebrafish model, human CHAF1A was constitutively overexpressed in NCCs using the pDest‐sox10:CHAF1A‐IRES‐EGFP‐pA2 construct and the parallel control construct pDest‐sox10:mcherry‐IRES‐EGFP‐pA2. Constructs were generated using FastCloning as previously described.^[^ ^72^ ^]^ To knock down CHAF1A expression, a previously validated TRIPZ lentiviral shRNA vector with a Tet‐inducible promoter and a TurboRFP reporter (GE Healthcare Dharmacon, Lafayette, CO) was used to transduce IMR32 and LAN5 cells.^[^ ^26^ ^]^ DOX was added to culture media at a final concentration of 1 or 2 µg mL^−1^ to induce CHAF1A overexpression or KD. NIH‐3T3 cells were transduced with HRAS‐overexpressing (pCDH‐CMV‐hRAS‐V12‐Bsd) and control lentiviruses (pCDH‐CMV‐Bsd) (Yang lab, BCM, Houston, TX). Inducible CHAF1A KD IMR32 cells were transduced with ODC1‐overexpressing (pHAGE‐EF1a‐ODC1‐stop‐mPGK‐Bsd) and control (pHAGE‐EF1a‐GFP‐stop‐mPGK‐Bsd) lentiviruses.

### In Vitro Functional Assays

Cell proliferation was determined using a Cell Counting Kit‐8 (CCK, Dojindo Molecular Technologies, Rockville, MD). Cell viability was determined by CellTiter‐Glo 2.0 (Promega, Madison, WI) following manufacturer protocol. Apoptosis was determined by Caspase‐Glo 3/7 Assay System (Promega) following manufacturer protocol. Luminescence signal was normalized by cell number. Cell motility and invasive capacity of SHEP and GIMEN cells were assessed using a Membrane Invasion Culture System as previously described.^[^ ^73^ ^]^ For cell cycle analysis, cells were fixed and stained using a Propidium Iodide Flow Cytometry Kit (Abcam). Data were analyzed using FlowJo (v7.6.1). Results are representative of at least two independent experiments. Neurite outgrowth upon changes in CHAF1A levels and RA/DFMO treatments was determined using an Olympus IX71 (Olympus, Center Valley, PA). Neurite length was quantified using Simple Neurite Tracer in Image J2,^[^ ^74^ ^]^ with 150–300 neurites quantified per treatment group. Data are the result of two independent experiments (mean ± SEM). For TUJ1 and TFAP2A staining in NB or NCCs, cells were fixed in cold methanol or paraformaldehyde (4%) and blocked in phosphate‐buffered saline containing goat serum (10%) and Triton X‐100 (0.2%), and incubated first with primary antibodies at 4 °C overnight (anti‐tubulin *β* III [TUJ1], 1:500, 801 201 or 1:1000, 801 202, Biolegend, San Diego, CA; TFAP2A, 1:200, 3B5, DSHB) and then with secondary antibodies (Alexa Fluor 488 Goat anti‐mouse IgG [1:500, 405 319, Biolegend]). Cells were observed under an Olympus IX71 with GFP channel. Zebrafish embryos were fixed in paraformaldehyde (4%) and immunostaining was performed as previously described.^[^ ^75^ ^]^ Primary antibodies: goat anti‐GFP (1:500, Abcam, 6673), mouse IgG2b anti‐Elavl3 (1:200, Invitrogen, A21271), mouse IgG1 anti‐pHH3 (1:1000, Abcam, ab14955), mouse IgG2a anti‐mCherry (1:500, GeneTex, GT844), and rabbit anti‐p150 CAF (1:250, Abcam, 126 625). Secondary antibodies at 1:500: 647 goat anti‐mouse IgG2b (Invitrogen, A21242), 647 goat anti‐mouse IgG1 (Invitrogen, A21240), 488 donkey anti‐goat (Invitrogen, A11055), 568 goat anti‐mouse IgG2a (Invitrogen, A21134), and 568 goat anti‐rabbit (Invitrogen, A11011). Embryos were imaged on an Olympus FV3000 laser scanning confocal microscope. ODC activity was measured by fluorescence‐based assay.^[^ ^76^ ^]^ One unit activity is defined as the change of fluorescence intensity per min. Data were normalized by protein amount and presented as the fold change relative to the control.

### Real‐Time qPCR and Western Blotting

Total RNA was isolated using an RNeasy Mini Kit (Qiagen, Germantown, MD) following the manufacturer's manual. The RNA was directly mixed with reagents supplied in the QuantiTect SYBR Green RT‐PCR Kit (Qiagen) and subjected to one‐step RT‐PCR performed on a StepOnePlus™ Real‐Time PCR System (Thermo Fisher Scientific). Available primer sequences are listed in Table S5, Supporting Information. Other primers were predesigned from KiCqStart SYBR Green, Sigma. Western blotting was performed as described previously.^[^ ^26^ ^]^ Primary antibodies: CHAF1A (1:1000, Abcam, 126 625), Cell Cycle Regulation Antibody Sampler Kit (1:500 or 1:1000, Cell Signaling, 9932), RAS (1:1000, cell signaling, 3965), and MYCN (1:500, cell signaling, 9405), ODC1 (1:400, Novus Biologicals, NBP2‐32887), HA Tag (1:2000, Abcam, ab9110), CypB (1:1000, 20 361, or 1:500, Santa Cruz Biotechnology, 130 626), *β*‐actin (1:10000, Sigma, A5316), and GAPDH (1:10 000, Proteintech, 10494‐AP). Secondary antibodies (LI‐COR Biosciences): IRDye 680RD donkey anti‐goat IgG (926‐68074), IRDye 680RD Goat anti‐mouse IgG (NC0809365), and IRDye 800CW Goat anti‐Rabbit IgG (926‐32211). Membranes were scanned on Odyssey Infrared Imaging System (LI‐COR Biosciences).

### Human NC Differentiation Model

For NC induction, hESC cells were digested with EDTA solution (2 mм) and seeded on Geltrex‐coated surfaces in induction medium (DMEM/F12 containing B27 supplement [2%, Fisher Scientific], 1 × Glutamax [Gibco]). The ROCK inhibitor Y‐27632 (10 µм, Tocris Bioscience) to stabilize hESC cells was added on Day (−1) and removed on Day (0). CHIR 99 021 (3 µм, 4423, Tocris Bioscience) was added on Day (0) to activate WNT signaling and was replenished daily until Day 5. On Day 5, the cells were harvested and TFAP2A immunofluorescence staining was performed. For NC differentiation, cells were treated with CHIR 99 021 for 5 days to induce NC formation and then plated at 20 000–80 000 cells cm^−1^ on a Geltrex‐coated surface in the absence or presence of DOX (2 µg mL^−1^). After 48 h, the induction medium was replaced with terminal differentiation medium (DMEM/F12 with B27 supplement [2%, Fisher Scientific], 1 × Glutamax [Gibco], and RA [500 nm, Sigma, R2625]). The media was subsequently replenished every 2 days. Cells were harvested 10–11 days post RA induction and TUJ1 immunostaining was performed.

### Zebrafish Model

For single cell dataset analysis, publicly available single cell RNAseq data from 48–50 hpf and 68–70 hpf zebrafish embryos (GEO accession: GSE152906) were analyzed as described using Seurat v3.1.1 software package for R.^[^ ^77^, ^78^ ^]^ Plots were generated using the FeaturePlot command. For HCR, probes against zebrafish *chaf1a* (NM_0 010 45013.2), *sox9b* (NM_131 644.1), *crestin* (AF195881.1), *mycn* (NM_212 614.2), and *elavl3* (NM_131 449) transcripts were generated and purchased from Molecular Instruments, Inc. The HCR protocol was performed as described.^[^ ^79^ ^]^ Zebrafish microinjections and microscopic imaging were performed in accordance with the guidelines of the Rice University Institutional Animal Care and Use Committee (protocol 1 143 754). CHAF1A‐overexpressing and parallel control clones were constructed as described above. Construct clones were screened by restriction digest and sequencing before microinjection into 1‐cell stage AB wild‐type embryos at a concentration of 50 pg construct along with 75 pg transposase mRNA, as described previously.^[^ ^80^ ^]^ The zebrafish embryos were sorted the next day for eGFP fluorescence and fixed in paraformaldehyde (4%) at 32 hpf. To observe spatial protein expression, embryos were stained with goat anti‐GFP (Abcam, ab6673), mouse IgG2b anti‐Elavl3 (Thermo Fisher Scientific, A‐21271), mouse IgG2a anti‐mCherry (Genetex, GT844), rabbit anti‐p150 CAF1 (abcam, ab126625), or mouse anti‐Histone H3 (phospho S10) (Abcam, ab14955), as described above. Whole mount embryos were embedded in glycerol (100%) and imaged on an Olympus FV3000 laser scanning confocal microscope using an Olympus UCPLFLN20x medium working distance objective. The Z‐stacks acquired were depicted as maximum‐intensity projections using Fluoview software and exported as tiff image files. Double positive clones (GFP+/GOI+ cells; GFP+/mCherry+ or GFP+/CHAF1A+) were detected, and their coincidence with Elavl3 or pHH3 was counted.

### NB Mouse Model

Animal studies were approved by the Institutional Animal Care and Use Committee of Baylor College of Medicine, Houston TX (AN4810 and AN7089). An orthotopic xenograft model of human NB was generated as described previously.^[^ ^50^ ^]^ Briefly, an inoculum of 10^6^ tumor cells in 0.1 mL PBS was injected under the renal capsule of 5 to 7‐week‐old female athymic NCr nude mice (Taconic, Hudson, NY). To determine whether CHAF1A promotes tumorigenesis, the mice were fed a control or DOX‐containing (0.625 g kg^−1^) diet for 5 weeks (ENVIGO, Indianapolis, IN). The mice were then sacrificed, and the tumor incidence or weight recorded. To determine the anti‐tumor activity of RA and DFMO, NCr nude mice (7 weeks old, female) were implanted with LAN5 luc cells which overexpress luciferase gene (10^6^ per mouse). Ten days after implantation, tumor engraftment was assessed via bioluminescence imaging (Xenogen IVIS 100 System, Caliper Life Sciences, MA). Mice were then divided into four groups: CTRL (1% methylcellulose, p.o., b.i.d., 5 days per week), 13‐*cis*‐RA (p.o., 40 mg kg^−1^ b.i.d., 5 days per week), DFMO (2% in sterile water, replaced weekly), and combination. Three weeks after treatment, mice were euthanized, and tumor weights recorded. To determine the anti‐tumor effects of RA upon genetic depletion of CHAF1A, NCr nude mice (7 weeks old, female) were implanted with LAN5 shCTRL, and shCHAF1A cells (10^6^ per mouse). Ten days after implantation, mice were treated with either vehicle (1% methylcellulose, p.o., b.i.d., 5 days per week) or 13‐*cis*‐RA (p.o., 40 mg kg^−1^ b.i.d., 5 days per week). Three weeks after treatment, mice were euthanized, and the tumor weights recorded. Assessment of tumor apoptosis was performed as previously described.^[^ ^81^ ^]^ Briefly, paraffin embedded tumor sections were blocked with horse serum (10%) and incubated with cleaved caspase‐3 antibody (1:400, Cell Signaling, 9661L) at 4 °C overnight. Sections were washed with PBS and incubated with biotinylated anti‐mouse (1:200, Vector Laboratories, Burlingame, CA, BA9200) and anti‐rabbit (1:200, Vector Laboratories, BA1000) antibodies at room temperature for 30 min, following incubation with 3,3′‐diaminobenzidine solution and counterstaining with hematoxylin (Fisher Scientific, 7211). Four representative fields per sample were taken by Olympus IX71 (×40). Cleaved caspase‐3 positive cells and total number of cells were counted in Image J2. Data are presented as percentage of cleaved caspase‐3 positive cells.

### Gene Expression Profiling and Analysis

CHAF1A was conditionally turned on for 0–96 h in SHEP cells or silenced for 5 or 10 days in IMR32 cells. Total RNA was extracted and processed by the random primed RT‐IVT‐RT method using the GeneChip WT cDNA Synthesis and Amplification Kit (Affymetrix, Santa Clara, CA). cDNA was synthesized from 200 ng total RNA by reverse transcription using T7 promoter‐(N6) oligonucleotides as primers. The cDNA was then fragmented and hybridized for 17 h at 45 °C to GeneChip Human Gene 2.0 ST Arrays (Affymetrix). Raw microarray CEL files were processed in R (3.4.1)^[^ ^82^ ^]^ using rma function in the biocLite Affy package^[^ ^83^ ^]^ and the hugene.2.0st annotation. shCHAF1A CEL files were converted in R (3.5.2) using rma function in BiocManager Affy package (1.60.0) and Affymetrix Human Genome U133 Plus 2.0 Array annotation. Maximum probe values were selected for each gene. FDR was computed using a Benjamini–Hochberg corrected two‐sided homoscedastic *t*‐test. *CHAF1A* signature was generated by filtering differentially expressed genes according to absolute fold change ≥ 1.25 and FDR < 0.1 in SHEP cells (CHAF1A ON versus OFF) and IMR32 cells (CHAF1A KD versus CTRL) (Table S2, Supporting Information). To further refine genes of clinical relevance, the authors integrated the *CHAF1A* signature with two transcriptomic clinical cohorts of NB patients (cohort 1 and 2). Within each of the cohorts, they assessed the Pearson Correlation Coefficient of every *CHAF1A* signature gene with *CHAF1A*. They considered a correlation to be significant for FDR < 0.1, and imposed the additional constraint that the correlation direction should match the direction of change of a gene in the *CHAF1A* signature. Following this method, multiple gene signatures were derived (Table S2, Supporting Information): CHAF1A OE + patient = genes differentially expressed in SHEP cells (CHAF1A ON 96 h versus OFF, absolute fold change ≥ 1.25 and FDR < 0.1) and correlated with *CHAF1A* expression in patient cohorts 1 or 2 (FDR < 0.1); CHAF1A KD1 + patient = genes differentially expressed in IMR32 cells (CHAF1A KD 5 days versus CTRL, absolute fold change ≥ 1.25 and FDR < 0.1) and correlated with *CHAF1A* expression in patient cohorts 1 or 2 (FDR < 0.1); CHAF1A KD2 + patient = genes differentially expressed in IMR32 cells (CHAF1A KD 10 days versus CTRL, absolute fold change ≥ 1.25 and FDR < 0.1) and correlated with *CHAF1A* expression in patient cohorts 1 or 2 (FDR < 0.1); CHAF1A KD3 + patient = genes differentially expressed in IMR32 cells (CHAF1A KD 5 and 10 days versus CTRL, absolute fold change ≥ 1.25 and FDR < 0.1) and correlated with *CHAF1A* expression in patient cohorts 1 or 2 (FDR < 0.1). The *MYCN* activity signature was derived from the 157 gene overlapping cell line and patient signature in Valentijn et al.^[^ ^84^ ^]^ For gene ontology (GO) analysis, genes with at least a 1.25 fold change and FDR of less than 0.1 for between‐group comparisons were analyzed by over‐representation analysis (ORA) using the MsigDB GO gene set. A pathway was considered enriched for FDR < 0.05. To analyze signature correlations, in each cohort, a z‐score was computed per patient sample for individual genes. Next, for each individual signature, the authors computed the activity score for each patient sample by adding the z‐scores of up‐regulated genes and subtracting the z‐score of downregulated genes.^[^ ^85^ ^]^ For each combination of gene signatures, the activity scores were plotted on the *x* and *y* axes and the Pearson Correlation Coefficient and *p*‐value were calculated using the Python scientific library, with significance achieved at *p* < 0.05.

### Metabolomics Profiling and Targeted Polyamine Analysis

SHEP cells with CHAF1A induction for 0, 24, and 72 h were used for global metabolomics (*n* = 5 replicates per group). Metabolites were prepared and analyzed by Metabolon Inc. (DiscoveryHD4 Metabolic Platform, *n* = 545 compound library, Durham, NC). Welch's two‐sample *t*‐test was performed to compare the difference in metabolite levels between groups; *p*‐values ≤ 0.05 were considered statistically significant. Metabolite set enrichment was implemented using the GSEA program (v2.24)^[^ ^71^ ^]^ with Metabolon metabolite sub‐pathways as reference. The GSEA program was run with 10 000 randomized metabolite sets for estimation of statistical significance (FDR < 0.25). The signal‐to‐noise metric (Z‐score) between the two phenotypes was used for ranking. For targeted polyamine analysis, samples were prepared as follows. Cell pellets (*n* = 4 per group) were resuspended in methanol:water (4:1) with internal standards (ISTD). Samples were then extracted by choloroform:water (3:1) solution, followed by phase separation, drying and removal of proteins and lipids. Mouse liver tissues were used as quality controls along with cell pellets. Polyamines were extracted using the liquid‐liquid extraction method previously described.^[^ ^86^, ^87^, ^88^ ^]^ The extracted samples were resuspended into methanol‐water (50:50 v/v) and subjected to chromatographic separation in hydrophilic interaction chromatography separation mode with XBridge Amide column (3.5 µm, 4.6 × 100 mm, ESI‐positive ionization, Waters, Milford, MA). Formic acid (0.1%) in water and acetonitrile were used as mobile phase A and B, respectively, as previously described.^[^ ^89^, ^90^ ^]^ The sample injection volume was 10 µL. The data were acquired via multiple reaction monitoring using a 6495 Triple Quadrupole mass spectrometry coupled to an HPLC system (Agilent Technologies, Santa Clara, CA) through Agilent Mass Hunter Data Acquisition Software (v10.1).^[^ ^91^ ^]^ The acquired mass spectra were analyzed and integrated of each targeted compound peak using Agilent Mass Hunter Quantitative Analysis Software (v10.1). The quantified peak areas were normalized with a spiked internal standard, and the data were log_2_‐transformed. In IMR32 shCHAF1A cells the differential compounds were determined by Benjamini‐Hochberg corrected false discovery rate (FDR < 0.25). In LAN5 and IMR32 cells treated with RA and DFMO, the differential compounds were determined by two‐way ANOVA with original FDR method of Benjamini and Hochberg (FDR < 0.05).

### Chromatin Immunoprecipitation (ChIP)

For MYCN ChIP, single‐step ChIP‐qPCR was performed by preparing nuclei from 1 × 10^7^ fresh formaldehyde fixed cells. Briefly, cells were cross‐linked using formaldehyde to a final concentration of 1% in complete culture media, and reaction was stopped using Glycine 5′ (0.125 м) at 25 °C. Nuclei were prepared by resuspending cells in cell lysis buffer (Pipes pH 8–5 mм, KCl ‐ 85 mм, NP40 ‐ 0.5%, PMSF ‐ 1 mм, Roche complete protease inhibitor) and after 4000 g centrifugation, modified RIPA‐sonic buffer (Tris‐HCL pH 8–50 mм, NaCl ‐ 150 mм, SDS ‐ 0.5%, NP40 ‐ 1%, PMSF ‐ 1 mм, Roche complete protease inhibitor) was added to perform nuclei lysis. Nuclei were then sonicated (Bioruptor‐plus Diagenode) to shear genomic DNA (150–300 bp). A small aliquot of sonicated material was put aside (Input sample), and the remaining sample (SDS diluted to 0.2%) immunoprecipitated using MYCN ChIP‐grade antibody (5 µg, Santa‐cruz B8.4.B). Rec‐sepharose Protein A (Invitrogen) were used to immobilize immuno‐complexes, and several washes were performed as follows: 6×RIPA‐wash buffer (Tris‐HCL pH 8–50 mм, NaCl ‐ 150 mм, SDS ‐ 0.1%, NP40 ‐ 1%, PMSF ‐ 1 mм), 2× high salt buffer (Tris‐HCL pH 8–50 mм, LiCl_2_ ‐ 250 mм, NP40 ‐ 1%, PMSF ‐ 1 mм), and 2× TE buffer. Immuno‐complexes were de‐crosslinked by using RNAse‐A (10 µg – 37°C 60’) and proteinase K (114 µg Roche) for 6 h at 65 °C. Finally, immunoprecipitated and input DNA samples were purified using phenol/chloroform and ethanol precipitation techniques. Purified DNA samples were then analyzed by qPCR using ΔΔCT method, and all the primers used are presented in Table S5, Supporting Information. For HA‐CHAF1A ChIP, a dual‐step ChIP‐qPCR was performed by preparing nuclei from 2 × 10^7^ cells that were pre‐incubated for 45 s at 25 °C with 2 mм di‐succinyl glutarate before formaldehyde fixing. All the steps performed after formaldehyde cross‐linking are reported in the single‐step ChIP‐qPCR above. ChIP‐grade HA antibody (Abcam, ab9110) was used to immunoprecipitate HA‐tagged CHAF1A; normal IgG rabbit (Millipore, 12–370) was used as negative control.

### Statistical Analysis

The analysis of gene expression and metabolic data is described in details in the individual Experimental sections. Cell viability data were normalized to control and expressed as percentages of control. Cell apoptosis, mRNA and protein expression, ChIP qPCR and ODC activity data were normalized to control and expressed as fold change. Data were mean ± SD or mean ± SEM with sample size *n* = biological replicates unless specified in the figure legend. For two‐group comparisons, the authors assumed a normal distribution and performed two‐sided unpaired *t*‐test. For multiple‐group comparisons, they used one‐way or two‐way ANOVA with Sidak's (compare selected means), Dunnett's (compare every mean with a reference mean), or Tukey's (compare every mean with every other mean) multiple comparisons test. For animal studies, the difference in tumor incidence between groups was computed by Fisher's exact test. The difference in tumor weights was computed by Mann–Whitney test. *p* values < 0.05 were considered statistically significant. Analyses were performed using GraphPad Prism (v7).

## Conflict of Interest

The authors declare no conflict of interest.

## Author Contributions

Conceptualization: L.T., C.C., P.S., G.P., R.J.P., R.A.U., and E.B.; methodology: L.T., M.M.‐S., R.I.‐G.‐P., G.M., N.A.D., Y.S.O., I.P., J.J.K., A.H.M.K., Y.Z., S.A.V., R.A.U., and E.B.; formal analysis: B.Z., T.P., J.H., C.C., and P.S.; investigation: L.T., M.M.‐S., R.I.‐G.‐P., G.M., N.A.D., B.E.H., Y.S.O., and E.B.; writing—original Draft: L.T., R.I.‐G.‐P., G.M., Y.S.O., B.Z., T.P., C.C., R.A.U., and E.B.; writing—review and editing: L.T., R.I.‐G.‐P., G.M., N.A.D., C.C., R.A.U., R.J.P., G.P., and E.B.; supervision: N.P., C.C., P.S., G.P., R.J.P., R.A.U., and E.B.

## Supporting information

Supporting Information

Supplemental Table 1

Supplemental Table 2

Supplemental Table 3

Supplemental Table 4

Supplemental Table 5

## Data Availability

The data that supports the findings of this study are available in the supplementary material of this article.
