# Supplementary material for: CHAF1A Blocks Neuronal Differentiation and Promotes Neuroblastoma Oncogenesis via Metabolic Reprogramming
Source: Adv Sci (Weinh). 2021 Aug 8;8(19):2005047. doi: 10.1002/advs.202005047 (PMC8498874; doi:10.1002/advs.202005047)
Supplement: Supplementary file 1 — Supporting Information [file ADVS-8-2005047-s001.pdf]

WILEY-VCH

© 2021 Wiley-VCH GmbH

ADVANCED SCIENCE
Open Access

Supporting Information

for *Adv. Sci.*, DOI: 10.1002/advs.202005047

CHAF1A Blocks Neuronal Differentiation and Promotes Neuroblastoma Oncogenesis via Metabolic Reprogramming

*Ling Tao, Myrthala Moreno-Smith, Rodrigo Ibarra-García-Padilla, Giorgio Milazzo, Nathan A. Drolet, Blanca E. Hernandez, Young S. Oh, Ivanshi Patel, Jean J. Kim, Barry Zorman, Tajhal Patel, Abu Hena Mostafa Kamal, Yanling Zhao, John Hicks, Sanjeev A. Vasudevan, Nagireddy Putluri, Cristian Coarfa, Pavel Sumazin, Giovanni Perini, Ronald J. Parchem, Rosa A. Uribe, and Eveline Barbieri**

Supporting Information

CHAF1A Blocks Neuronal Differentiation and Promotes Neuroblastoma Oncogenesis via Metabolic Reprogramming

*Ling Tao, Myrthala Moreno-Smith, Rodrigo Ibarra-García-Padilla, Giorgio Milazzo, Nathan A. Drolet, Blanca E. Hernandez, Young S. Oh, Ivanshi Patel, Jean J. Kim, Barry Zorman, Tajhal Patel, Abu Hena Mostafa Kamal, Yanling Zhao, John Hicks, Sanjeev A. Vasudevan, Nagireddy Putluri, Cristian Coarfa, Pavel Sumazin, Giovanni Perini, Ronald J. Parchem, Rosa A. Uribe, and Eveline Barbieri**

Supplementary Figures:

Figure S1. CHAF1A and ODC1 protein expression in multiple NB cell lines.

Figure S2. CHAF1A promotes malignant cell phenotype.

Figure S3. CHAF1A blocks RA-induced NB differentiation.

Figure S4. CHAF1A blocks NC differentiation in hESC-NCC model.

Figure S5. CHAF1A-associated gene expression and metabolic changes.

Figure S6. Targeting polyamine metabolism reverses CHAF1A-mediated oncogenic functions.

Figure S7. Functional Relationship between CHAF1A and MYCN.

Supplementary Tables (attached separately):

Table S1. GSEA Hallmark analysis of two patient cohorts.

Table S2. Gene expression and signatures.

Table S3. Metabolomics and enrichment analysis.

Table S4. Polyamine analysis.

Table S5. qPCR and ChIP-qPCR primers.

Figure Legends

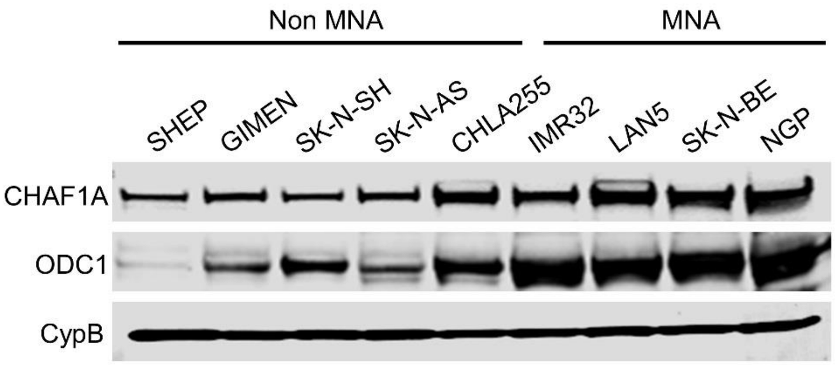

Figure S1. CHAF1A and ODC1 protein expression in multiple NB cell lines. CypB is used as loading control. Non MNA = Non MYCN-amplified NB cells; MNA = MYCN-amplified NB cells.

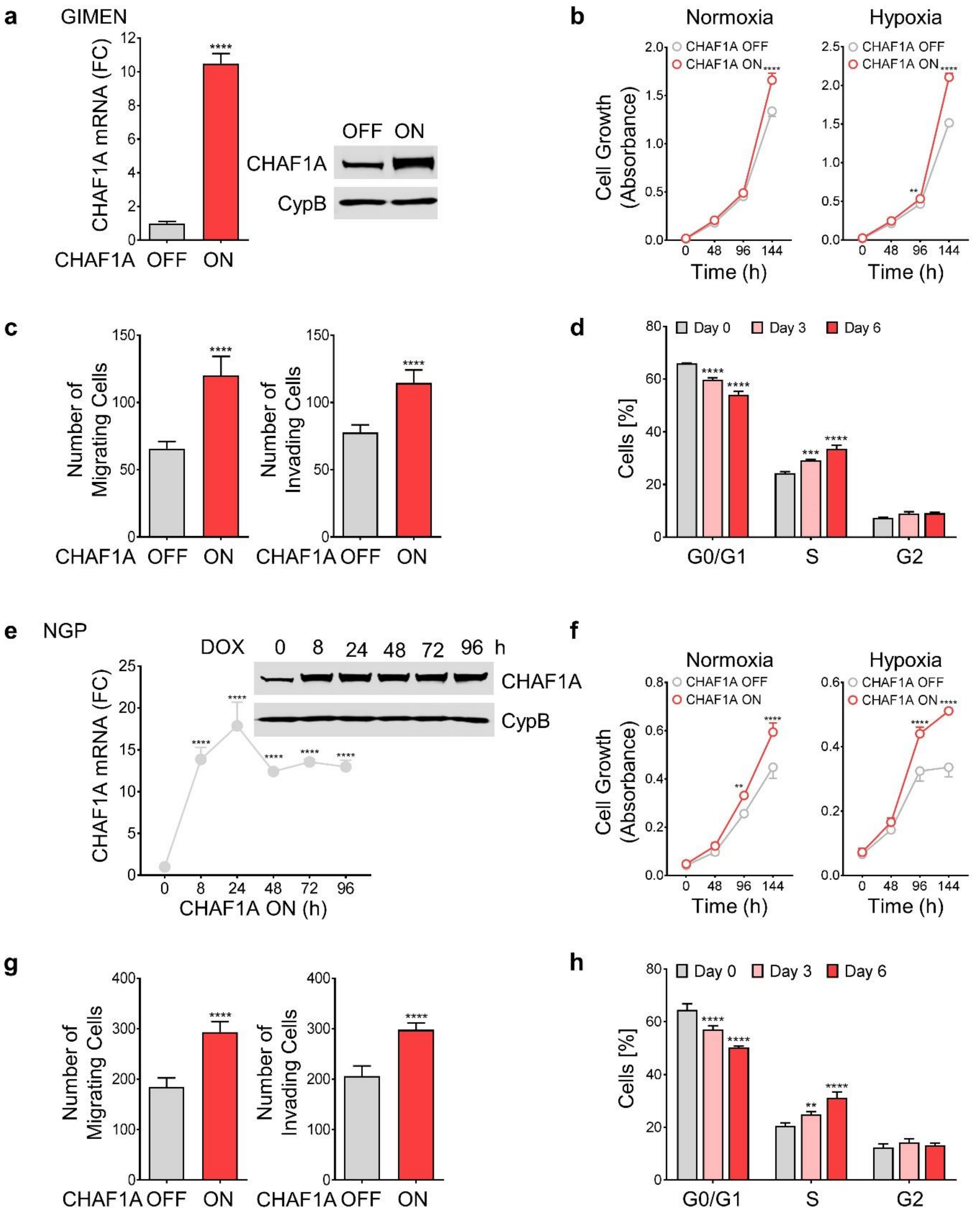
a
GIMEN
CHAF1A mRNA (FC)
CHAF1A OFF ON
OFF ON
CHAF1A
CypB
b
Normoxia
Hypoxia
CHAF1A OFF
CHAF1A ON
Cell Growth (Absorbance)
Time (h)
c
Number of Migrating Cells
Number of Invading Cells
CHAF1A OFF ON
d
Day 0
Day 3
Day 6
Cells [%]
G0/G1
S
G2
e
NGP
DOX 0 8 24 48 72 96 h
CHAF1A
CypB
CHAF1A mRNA (FC)
CHAF1A ON (h)
f
Normoxia
Hypoxia
CHAF1A OFF
CHAF1A ON
Cell Growth (Absorbance)
Time (h)
g
Number of Migrating Cells
Number of Invading Cells
CHAF1A OFF ON
h
Day 0
Day 3
Day 6
Cells [%]
G0/G1
S
G2

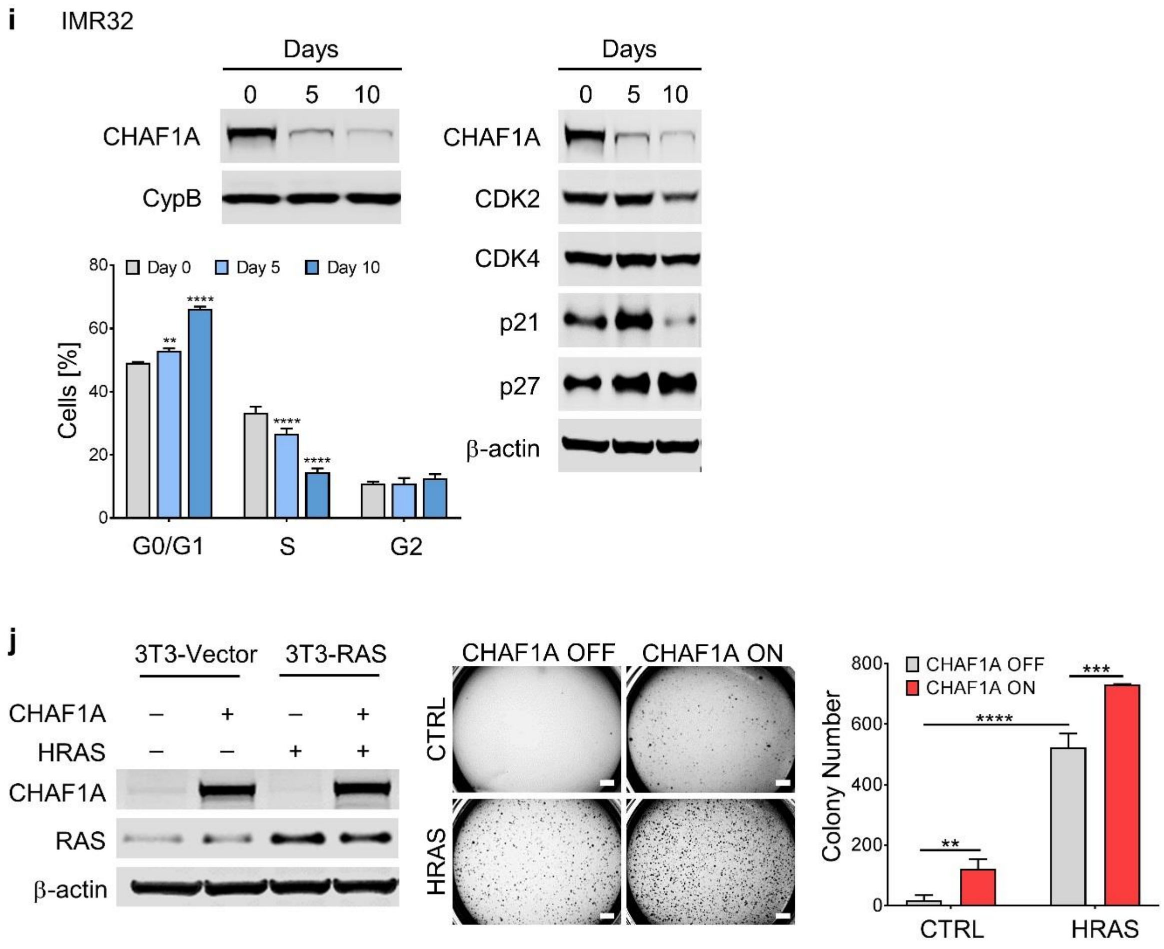

Figure S2. CHAF1A promotes aggressive cell phenotype. a) and e) Using a Tet-ON system, CHAF1A was turned on after DOX induction (1 μg mL^{-1}) in GIMEN (72 h) and NGP cells (0–96 h). CHAF1A overexpression was validated by qPCR and western blotting. Mean ± SD (n = 3); ****p < 0.0001; two-sided unpaired t-test (a) and one-way ANOVA with Dunnett's multiple comparisons test (b). b) and f) Proliferation assay (Counting Kit-8; absorbance at 450 nm) in GIMEN and NGP cells with or without CHAF1A overexpression. Cells were cultured in normoxic and hypoxic (1% O_2) conditions (0–144 h). Mean ± SD (n = 4); **p < 0.01, ****p < 0.0001; two-way ANOVA with Sidak's multiple comparisons test. c) and g) Migration and invasion analyses of GIMEN and NGP cells upon CHAF1A overexpression (48 h). Mean ± SD (n = 5–10); ****p < 0.0001; two-sided unpaired t-test. d) and h) Cell cycle analysis of GIMEN and NGP cells upon CHAF1A overexpression (0–6 days). Mean ± SD (n = 2–3); **p < 0.01, ***p < 0.001, ****p < 0.0001; two-way ANOVA with Dunnett's multiple comparisons test. i) Cell cycle analysis and cell cycle protein expression in IMR32 cells upon CHAF1A KD (0–10 days). Mean ± SD (n = 3); **p < 0.01, ****p < 0.0001; two-way ANOVA with Dunnett's multiple comparisons test. j) Left: HRAS and CHAF1A protein expression in NIH-3T3 cells;

β-actin was used as loading control. Middle and Right: Colony formation assay in NIH-3T3 fibroblast cells upon overexpression of CHAF1A, HRAS, or both. Images were taken by an Olympus SZX16. Colonies were counted using Quantity One software (v 4.6.9). Data are mean ± SD (n = 2–3); ** $p < 0.01$; *** $p < 0.001$; **** $p < 0.0001$; two-way ANOVA with Tukey's multiple comparisons test. FC = fold change.

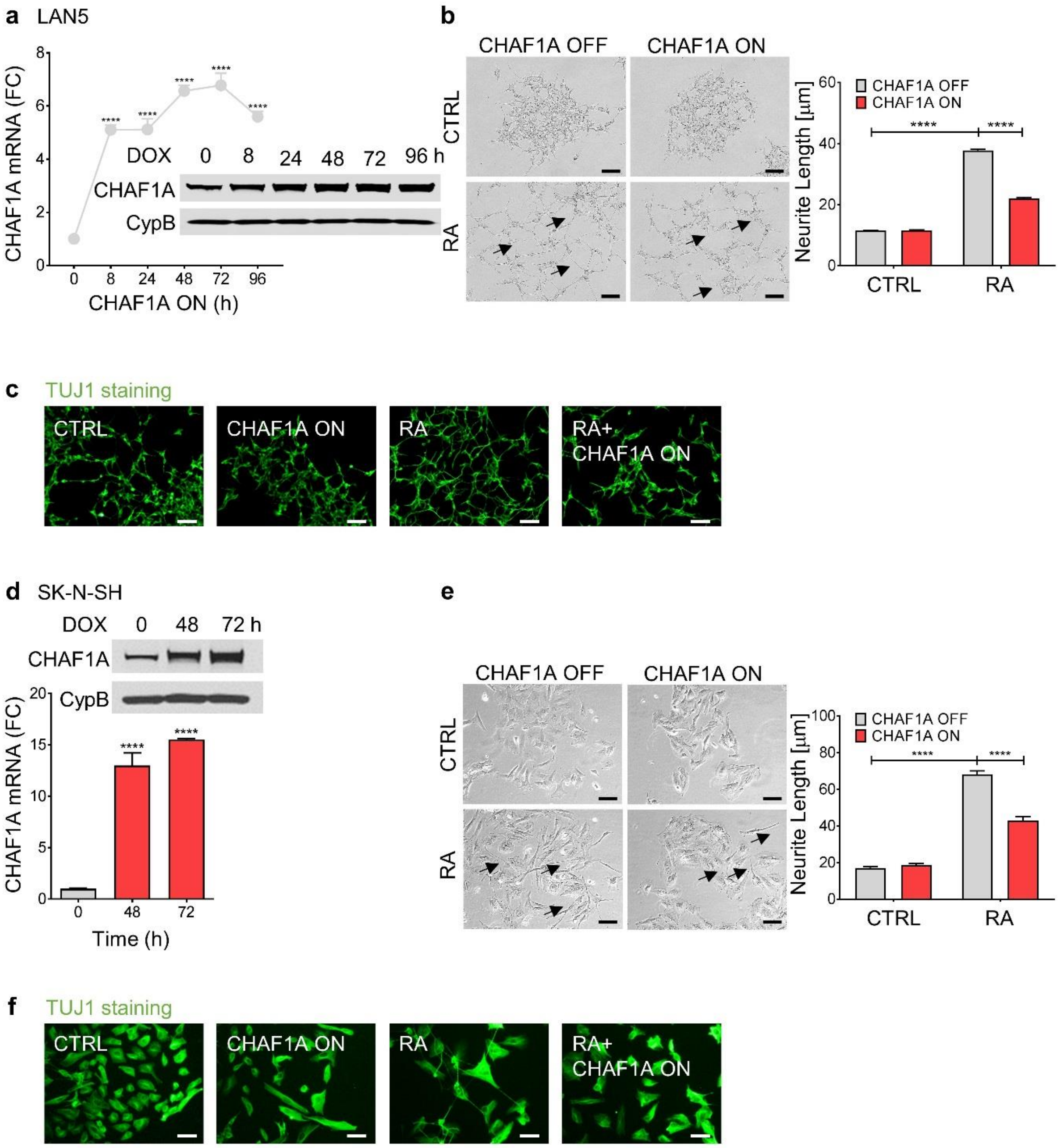
a LAN5
CHAF1A mRNA (FC)
CHAF1A ON (h)
DOX 0 8 24 48 72 96 h
CHAF1A
CypB
b
CHAF1A OFF
CHAF1A ON
CTRL
RA
Neurite Length [µm]
c TUJ1 staining
CTRL
CHAF1A ON
RA
RA+ CHAF1A ON
d SK-N-SH
DOX 0 48 72 h
CHAF1A
CypB
CHAF1A mRNA (FC)
Time (h)
e
CHAF1A OFF
CHAF1A ON
CTRL
RA
Neurite Length [µm]
f TUJ1 staining
CTRL
CHAF1A ON
RA
RA+ CHAF1A ON

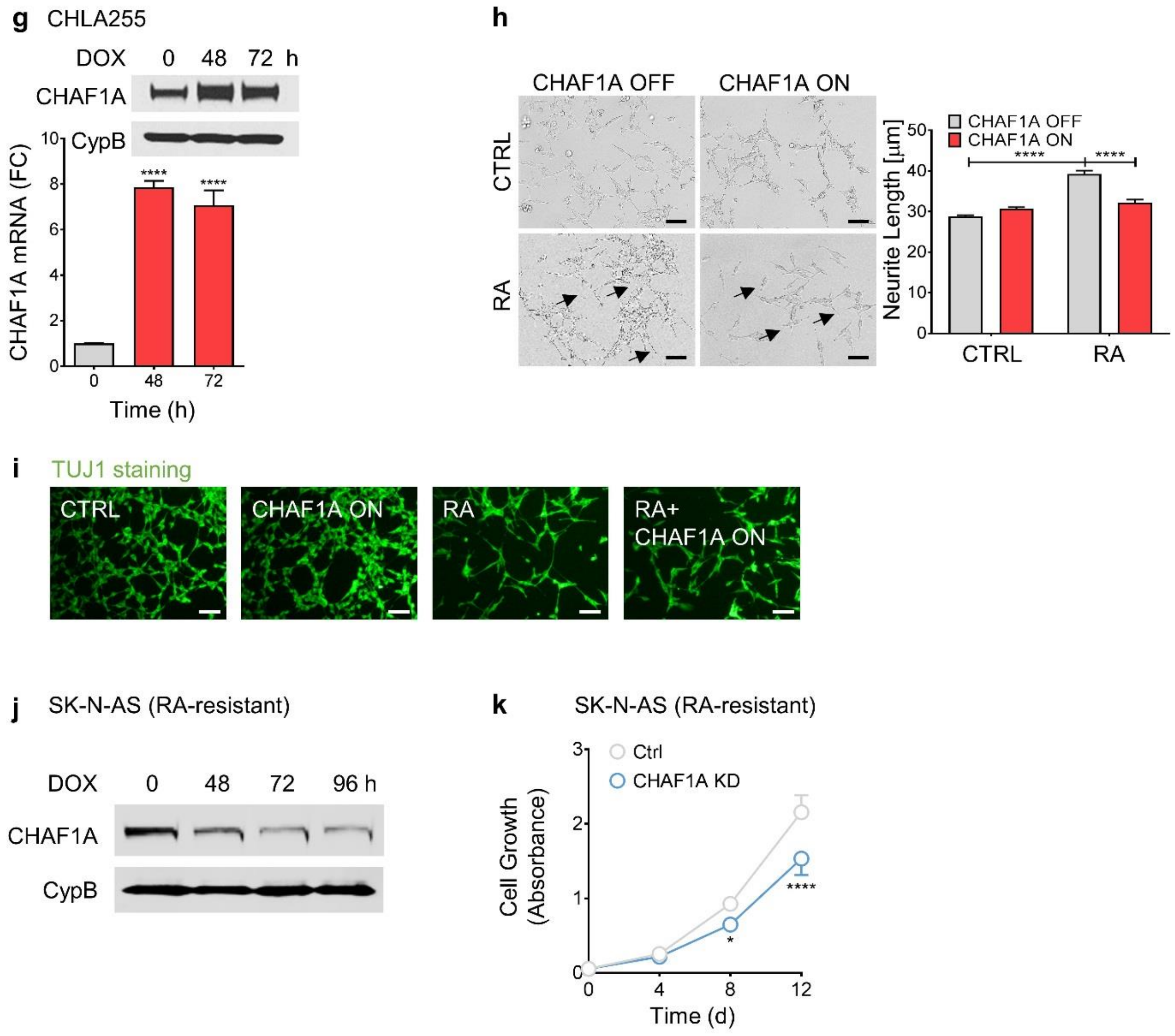

Figure S3. CHAF1A blocks RA-induced NB differentiation. a) CHAF1A mRNA and protein expression upon DOX (1 μg mL^{-1}) induction for 0–96 h in LAN5 cells. Mean ± SD (n = 3); ****p < 0.0001; one-way ANOVA with Dunnett's multiple comparisons test. b) Bright field images of neurite outgrowth and quantification of neurite length. Cells were pre-induced with DOX for 3 days before treatment with RA (10 μм) for 5 days. Neurite length was quantified by Image J2 and presented as mean ± SEM (n > 300, two biological replicates); ****p < 0.0001; two-way ANOVA with Tukey's multiple comparisons test. Scale bar = 50 μm. c) TUJ1 immunofluorescence staining. Scale bar = 50 μm. d) to i) Validation of CHAF1A overexpression, neurite quantification and TUJ1 staining in SK-N-SH (d to f) and CHLA255 (g to i) cells. Gene expression is mean ± SD (n = 3); ****p < 0.0001; one-way ANOVA with Dunnett's multiple comparisons test. Neurite length was quantified by Image J2 and presented as mean ± SEM (n > 300, two biological replicates); ****p < 0.0001; two-way ANOVA with

Tukey's multiple comparisons test. Scale bar = 50 μm. j) CHAF1A protein expression upon DOX-induced CHAF1A KD in SK-N-AS cells (1 μg mL^{-1}, 0–96 h). k) Cell growth upon CHAF1A KD (0–12 days). Mean ± SD (n = 4); * $p < 0.05$, **** $p < 0.0001$; two-way ANOVA with Sidak's multiple comparisons test. FC = fold change.

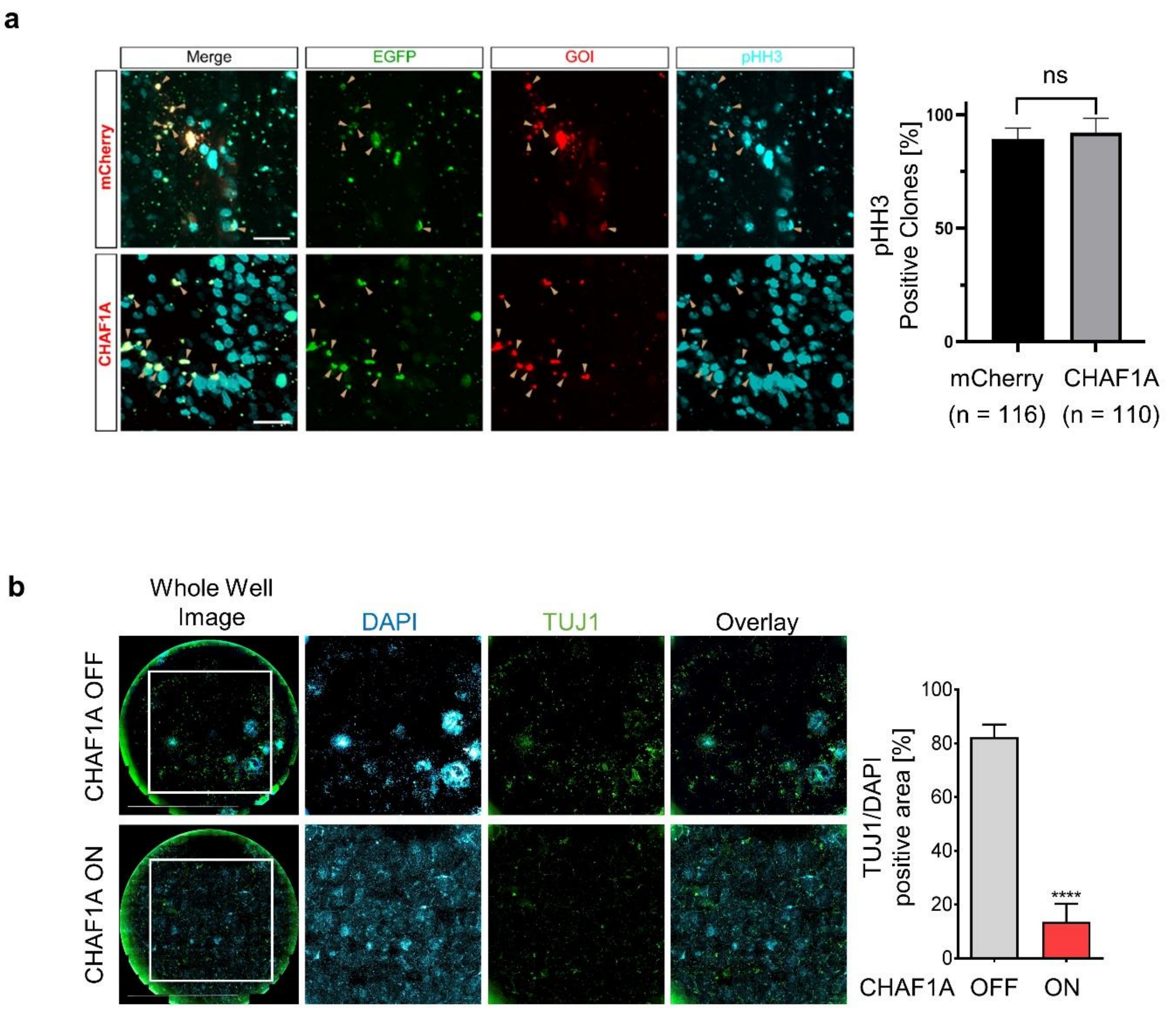

Figure S4. CHAF1A blocks NC differentiation in hESC-NCC model. a) Representative confocal images of the cranial region from *sox10*:mCherry-IRES-EGFP or *sox10*:chaf1a-IRES-EGFP injected embryos at 32 hpf. Markers: EGFP (green), gene of interest (GOI) either mCherry or CHAF1A (red), and proliferation marker pHH3 (S10 phosphorylated Histone H3) (cyan). Tan arrowheads indicate triple positive clones (GFP+/GOI+/pHH3+). Scale bars = 25 µm. Bar graph depicting the average percentage of double positive clones (GFP+/mCherry+ or GFP+/CHAF1A+) that also express pHH3+ antigen. Mean ± SEM (16 embryo per condition; mCherry n = 116 clones; CHAF1A n = 110 clones); two-sided unpaired t-test. b) Whole well imaging and quantification of TUJ1 in NCC-derived neurons with or without CHAF1A overexpression. NCCs were treated with RA (500 nM) with or without CHAF1A overexpression for 9 days before TUJ1 immunofluorescence staining. The whole well was imaged using Cytation5 and quantified using Image J2. Scale bar = 10 mm. Data are the mean ± SD (n = 4); **** $p < 0.0001$; two-sided unpaired t-test. ns = not significant ($p > 0.05$).

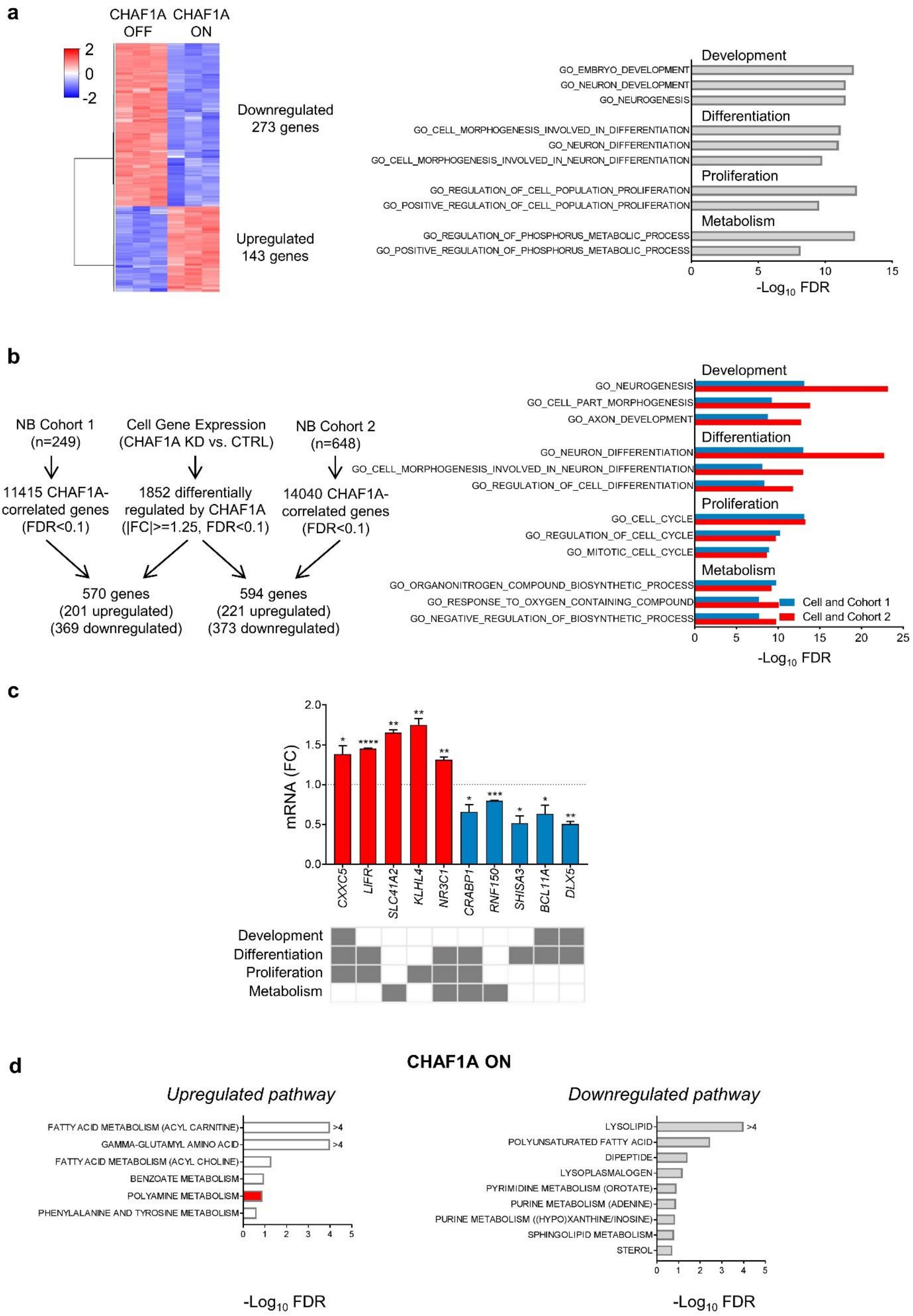
a
CHAF1A OFF
CHAF1A ON
2
0
-2
Downregulated 273 genes
Upregulated 143 genes
Development
GO_EMBRYO_DEVELOPMENT
GO_NEURON_DEVELOPMENT
GO_NEUROGENESIS
Differentiation
GO_CELL_MORPHOGENESIS_INVOLVED_IN_DIFFERENTIATION
GO_NEURON_DIFFERENTIATION
GO_CELL_MORPHOGENESIS_INVOLVED_IN_NEURON_DIFFERENTIATION
Proliferation
GO_REGULATION_OF_CELL_POPULATION_PROLIFERATION
GO_POSITIVE_REGULATION_OF_CELL_POPULATION_PROLIFERATION
Metabolism
GO_REGULATION_OF_PHOSPHORUS_METABOLIC_PROCESS
GO_POSITIVE_REGULATION_OF_PHOSPHORUS_METABOLIC_PROCESS
-Log10 FDR
b
NB Cohort 1 (n=249)
Cell Gene Expression (CHAF1A KD vs. CTRL)
NB Cohort 2 (n=648)
11415 CHAF1A-correlated genes (FDR<0.1)
1852 differentially regulated by CHAF1A (|FC|>=1.25, FDR<0.1)
14040 CHAF1A-correlated genes (FDR<0.1)
570 genes (201 upregulated) (369 downregulated)
594 genes (221 upregulated) (373 downregulated)
Development
GO_NEUROGENESIS
GO_CELL_PART_MORPHOGENESIS
GO_AXON_DEVELOPMENT
Differentiation
GO_NEURON_DIFFERENTIATION
GO_CELL_MORPHOGENESIS_INVOLVED_IN_NEURON_DIFFERENTIATION
GO_REGULATION_OF_CELL_DIFFERENTIATION
Proliferation
GO_CELL_CYCLE
GO_REGULATION_OF_CELL_CYCLE
GO_MITOTIC_CELL_CYCLE
Metabolism
GO_ORGANONITROGEN_COMPOUND_BIOSYNTHETIC_PROCESS
GO_RESPONSE_TO_OXYGEN_CONTAINING_COMPOUND
GO_NEGATIVE_REGULATION_OF_BIOSYNTHETIC_PROCESS
Cell and Cohort 1
Cell and Cohort 2
-Log10 FDR
c
mRNA (FC)
CXXC5
LIFR
SLC41A2
KLHL4
NR3C1
CRABP1
RNF150
SHISA3
BCL11A
DLX5
Development
Differentiation
Proliferation
Metabolism
d
CHAF1A ON
Upregulated pathway
FATTY ACID METABOLISM (ACYL CARNITINE)
GAMMA-GLUTAMYL AMINO ACID
FATTY ACID METABOLISM (ACYL CHOLINE)
BENZOATE METABOLISM
POLYAMINE METABOLISM
PHENYLALANINE AND TYROSINE METABOLISM
-Log10 FDR
Downregulated pathway
LYSOLIPID
POLYUNSATURATED FATTY ACID
DIPEPTIDE
LYSOPLASMALOGEN
PYRIMIDINE METABOLISM (OROTATE)
PURINE METABOLISM (ADENINE)
PURINE METABOLISM ((HYPO)XANTHINE/INOSINE)
SPHINGOLIPID METABOLISM
STEROL
-Log10 FDR

Figure S5. CHAF1A-associated gene expression and metabolic changes. a) Left: heat map of differentially expressed genes (DEGs, |log2 (fc)| >= 1, FDR < 0.1, z score scaled) between control (CHAF1A OFF) and CHAF1A-overexpressing SHEP cells (CHAF1A ON, 96 h). Right: GO pathway enrichment analysis of the overlapped genes (ranked by $–\text{Log}_{10}$FDR, FDR<0.05). FDR is computed by two-sided homoscedastic t-test corrected with Benjamini–Hochberg procedure. b) Overlap of DEGs, |(fc)| >= 1.25, FDR < 0.1) between control and CHAF1A-KD IMR32 cells (day 5) and *CHAF1A*-correlated genes (FDR < 0.1) in patient cohort 1 (n = 249) and 2 (n = 648). FDR is computed by two-sided homoscedastic t-test corrected with Benjamini–Hochberg procedure. c) qPCR validation of CHAF1A-associated genes that are involved in development, differentiation, proliferation and metabolism in IMR32 cells after CHAF1A KD for 5 days (mRNA fold change normalized to CTRL). Mean ± SD (n = 4); * $p < 0.05$; ** $p < 0.01$; *** $p < 0.001$; **** $p < 0.0001$; two-sided unpaired t-test. d) Metabolic pathway enrichment analysis in SHEP cells after CHAF1A induction (72 h, n = 5). Data are presented as $–\text{Log}_{10}$FDR (FDR < 0.05); Benjamini–Hochberg corrected two-sided homoscedastic t-test. The polyamine pathway is highlighted in red. FC = fold change.

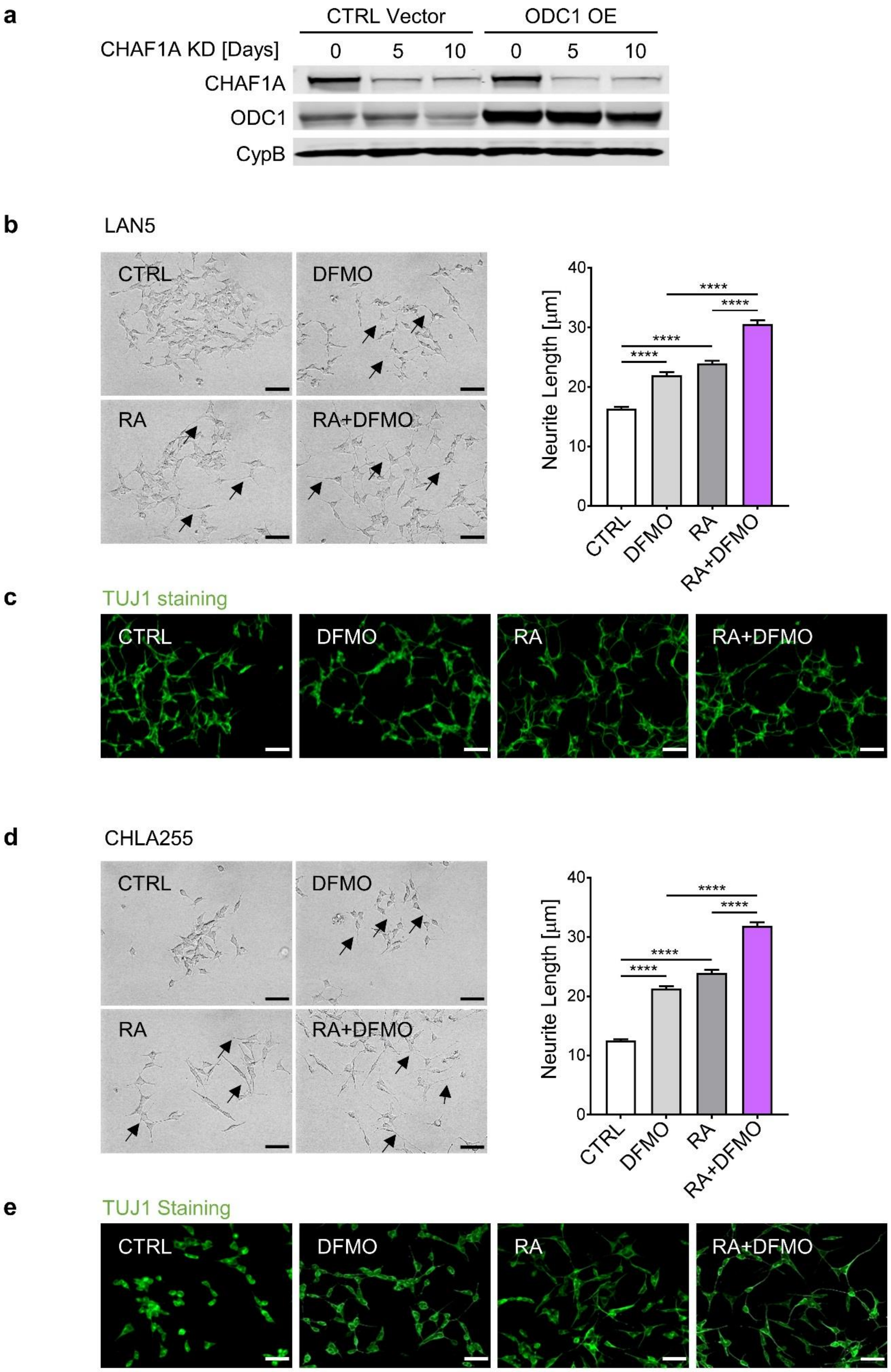
a
CTRL Vector
ODC1 OE
CHAF1A KD [Days]
0
5
10
0
5
10
CHAF1A
ODC1
CypB
b
LAN5
CTRL
DFMO
RA
RA+DFMO
Neurite Length [µm]
40
30
20
10
0
****
****
****
****
CTRL
DFMO
RA
RA+DFMO
c
TUJ1 staining
CTRL
DFMO
RA
RA+DFMO
d
CHLA255
CTRL
DFMO
RA
RA+DFMO
Neurite Length [µm]
40
30
20
10
0
****
****
****
****
CTRL
DFMO
RA
RA+DFMO
e
TUJ1 Staining
CTRL
DFMO
RA
RA+DFMO

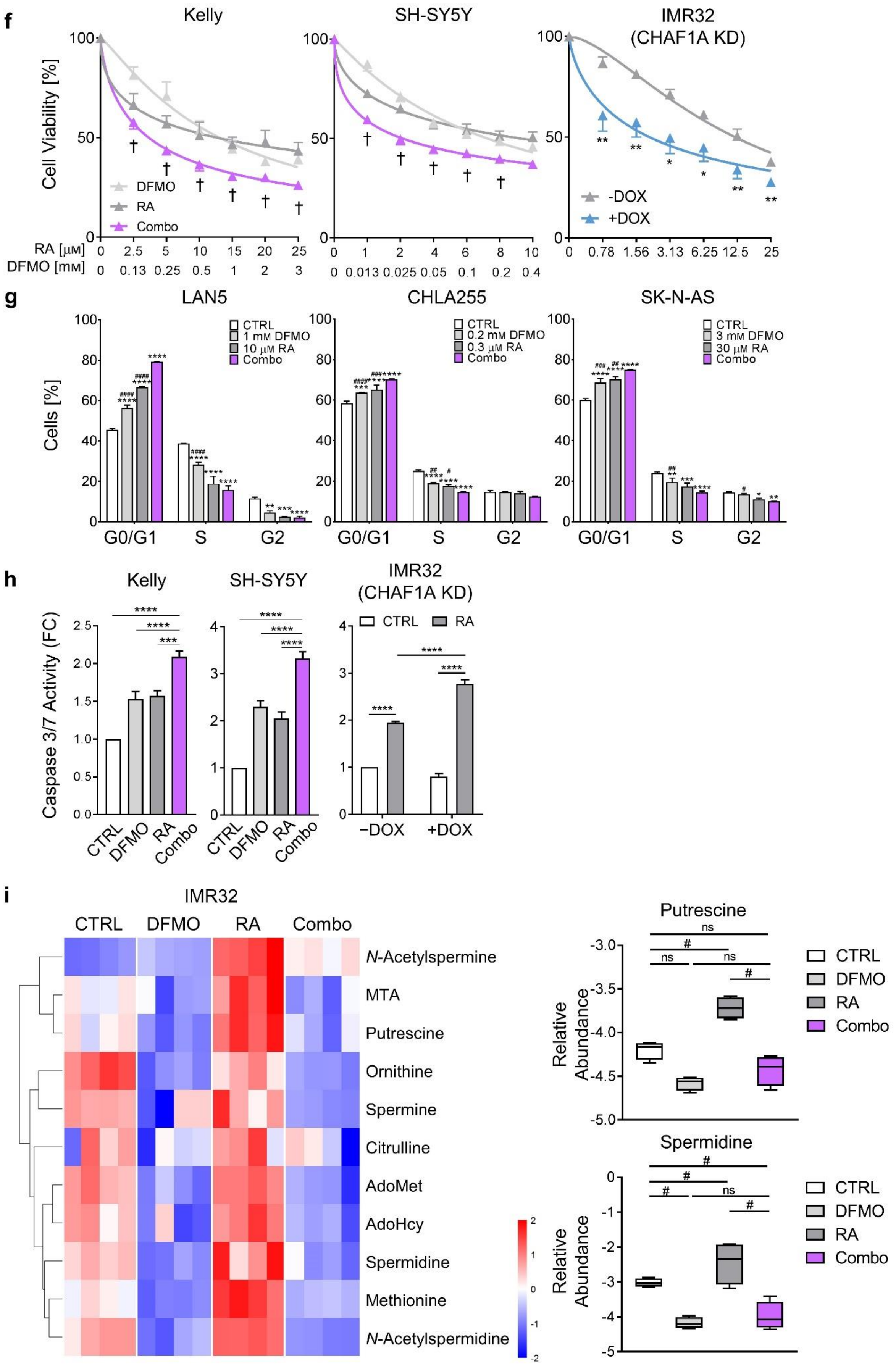
f
Kelly
SH-SY5Y
IMR32
(CHAF1A KD)
Cell Viability [%]
DFMO
RA
Combo
-DOX
+DOX
RA [μM]
DFMO [mM]
0 2.5 5 10 15 20 25
0 0.13 0.25 0.5 1 2 3
0 1 2 4 6 8 10
0 0.013 0.025 0.05 0.1 0.2 0.4
0 0.78 1.56 3.13 6.25 12.5 25
g
LAN5
CHLA255
SK-N-AS
Cells [%]
CTRL
1 mM DFMO
10 μM RA
0.2 mM DFMO
0.3 μM RA
3 mM DFMO
30 μM RA
Combo
G0/G1
S
G2
h
Kelly
SH-SY5Y
IMR32
(CHAF1A KD)
Caspase 3/7 Activity (FC)
CTRL
DFMO
RA
Combo
−DOX
+DOX
i
IMR32
CTRL
DFMO
RA
Combo
N-Acetylspermine
MTA
Putrescine
Ornithine
Spermine
Citrulline
AdoMet
AdoHcy
Spermidine
Methionine
N-Acetylspermidine
Putrescine
Spermidine
Relative Abundance
ns
#
CTRL
DFMO
RA
Combo

i (cont.)

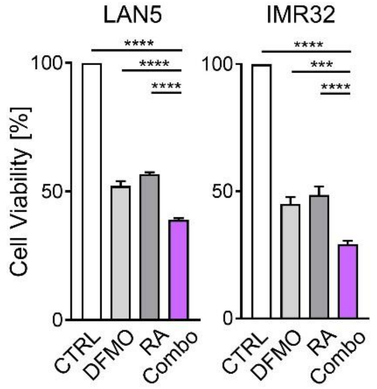

j

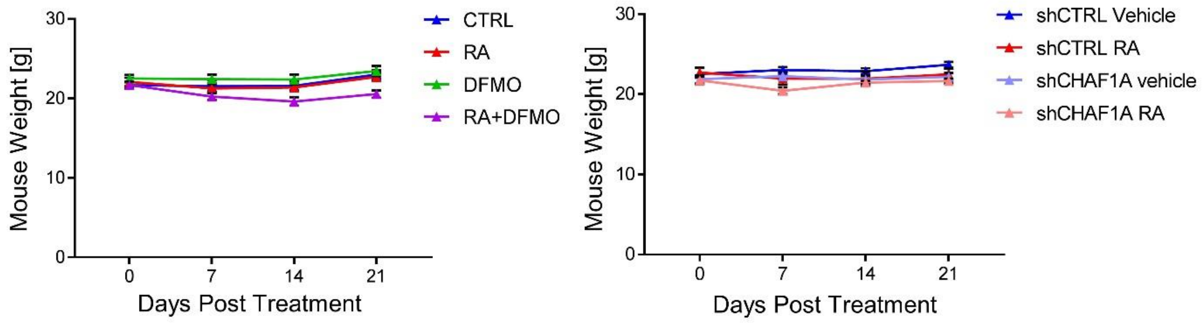

Figure S6. Targeting polyamine metabolism reverses CHAF1A-mediated oncogenic functions. a) Determination of CHAF1A and ODC1 protein expression in IMR32 cells with or without ODC1 overexpression (ODC1 ORF or vector CTRL) upon DOX (1 µg mL^{-1})-induced CHAF1A KD. b) and c) Neurite length and TUJ1 immunofluorescence staining in LAN5 cells treated with RA (5 µM) alone or in combination with DFMO (300 µM) for 5 days. Neurite length was quantified using Image J2. Data are mean ± SEM (n > 300); ****p<0.0001; one-way ANOVA with Tukey's multiple comparisons test. Scale bar = 50 µm. d) and e) Neurite length and TUJ1 immunofluorescence staining in CHLA255 cells treated with RA (0.3 µM) alone and in combination with DFMO (10 µM) for 72 h. Neurite length was quantified using Image J2. Data are mean ± SEM (n > 300); ****p<0.0001; one-way ANOVA with Tukey's multiple comparisons test. Scale bar = 50 µm. f) Cell viability of Kelly and SH-SY5Y cells treated with DFMO, RA alone or their combination (combo). Cell viability of IMR32 cells with conditional CHAF1A KD (DOX 1 µg mL^{-1}) in the presence or absence of RA. Data are the mean ± SD (n = 3). †synergy with CI < 1; *p < 0.05; **p < 0.01; two-sided unpaired t-test. g) Cell cycle analysis of LAN5, CHLA255 and SK-N-AS cells treated with RA alone and in combination with DFMO (IC_{50}). Data are mean ± SD (n = 2); *significance compared to CTRL (* p < 0.05,

** $p < 0.01$, *** $p < 0.001$, **** $p < 0.0001$); #significance compared to combo (# $p < 0.05$, ## $p < 0.01$, ### $p < 0.001$, #### $p < 0.0001$); two-way ANOVA with Tukey’s multiple comparisons test. h) Apoptosis of Kelly and SH-SY5Y cells treated with DFMO, RA or combo ($IC_{50–75}$). Apoptosis of IMR32 cells (-/+ DOX to induce CHAF1A KD) treated with RA (2 µM). Data are mean ± SD (n = 3); * $p < 0.05$, ** $p < 0.01$, *** $p < 0.001$, **** $p < 0.0001$; two-way ANOVA with Tukey's multiple comparisons test. i) Polyamine metabolites in IMR32 cells treated with DFMO (1 mM), RA (5 µM) or combo for 3 days (n = 4 per group). Metabolites with FDR < 0.05 in at least one comparison were shown in the heatmap (red = upregulated; blue = downregulated); two-way ANOVA with original FDR method of Benjamini and Hochberg. The relative abundance of putrescine and spermidine is presented in box and whiskers plots. # indicates FDR < 0.05. Viability of IMR32 and LAN5 cells (corresponding to Figure 5h) upon treatment of RA and/or DFMO is shown. Mean ± SD (n = 3); *** $p < 0.001$, **** $p < 0.0001$; one-way ANOVA with Tukey's multiple comparisons test. j) Mouse weights corresponding to study in Figure 5i and 5j. Mean ± SEM (n = 8–11). MTA = 5'-methylthioadenosine; AdoMet = *S*-(5'-Adenosyl)-*L*-methionine; AdoHyc = *S*-(5'-Adenosyl)-*L*- homocysteine. FC = fold change; ns = not significant.

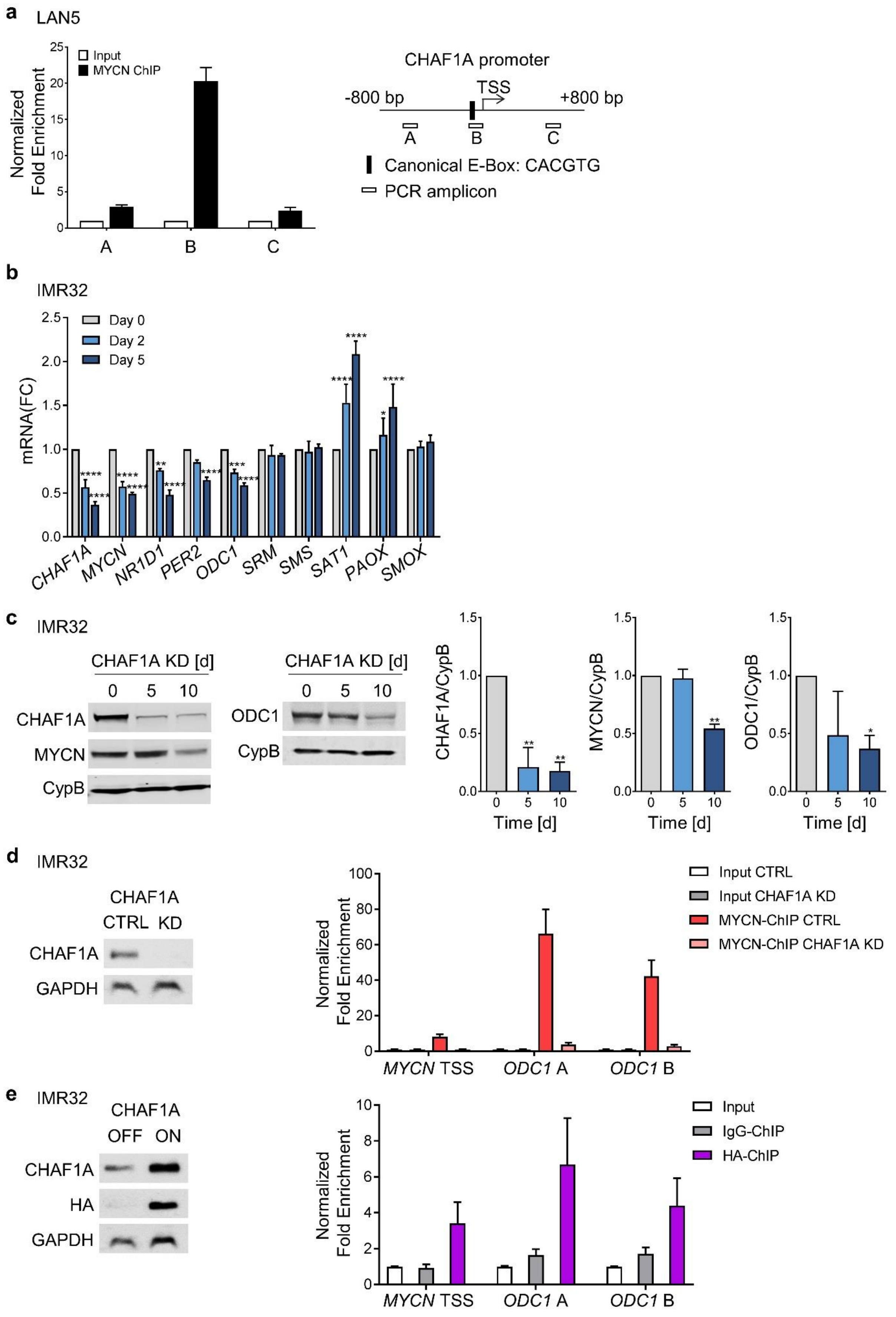
a
LAN5
Input
MYCN ChIP
Normalized Fold Enrichment
A
B
C
CHAF1A promoter
-800 bp
TSS
+800 bp
Canonical E-Box: CACGTG
PCR amplicon
b
IMR32
Day 0
Day 2
Day 5
mRNA(FC)
CHAF1A
MYCN
NR1D1
PER2
ODC1
SRM
SMS
SAT1
PAOX
SMOX
c
IMR32
CHAF1A KD [d]
0 5 10
CHAF1A
MYCN
CypB
ODC1
CHAF1A/CypB
MYCN/CypB
ODC1/CypB
Time [d]
d
IMR32
CHAF1A
CTRL KD
GAPDH
Input CTRL
Input CHAF1A KD
MYCN-ChIP CTRL
MYCN-ChIP CHAF1A KD
MYCN TSS
ODC1 A
ODC1 B
e
IMR32
CHAF1A
OFF ON
HA
Input
IgG-ChIP
HA-ChIP

f

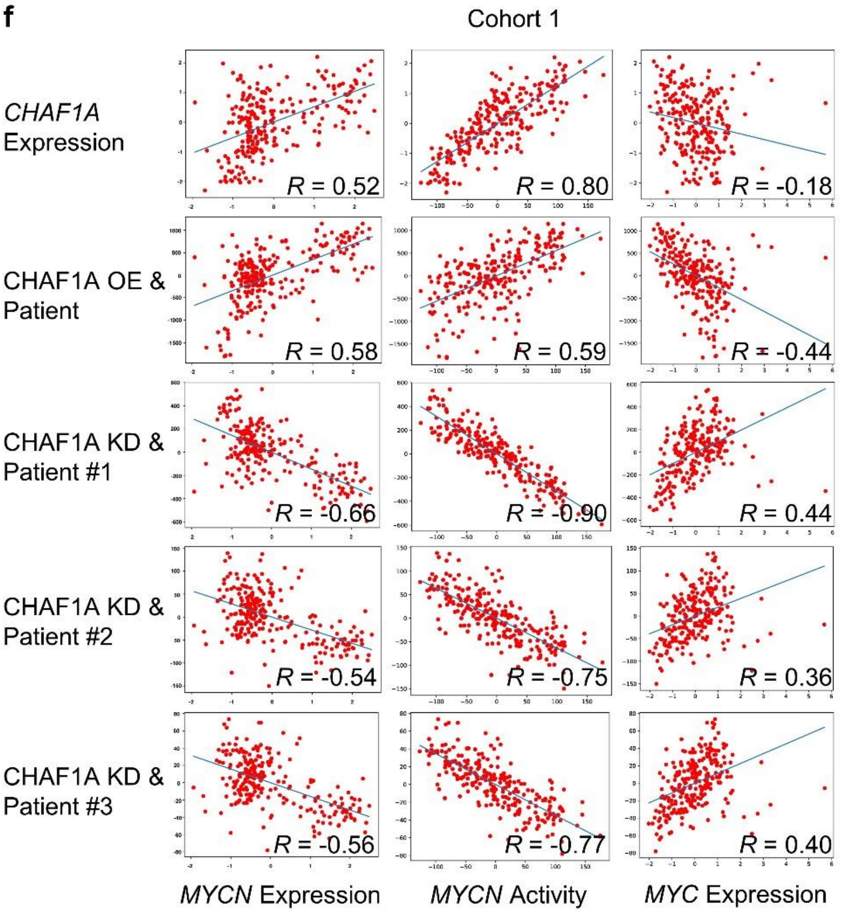
Cohort 1
CHAF1A Expression
R = 0.52
R = 0.80
R = -0.18
CHAF1A OE & Patient
R = 0.58
R = 0.59
R = -0.44
CHAF1A KD & Patient #1
R = -0.66
R = -0.90
R = 0.44
CHAF1A KD & Patient #2
R = -0.54
R = -0.75
R = 0.36
CHAF1A KD & Patient #3
R = -0.56
R = -0.77
R = 0.40
MYCN Expression
MYCN Activity
MYC Expression

g

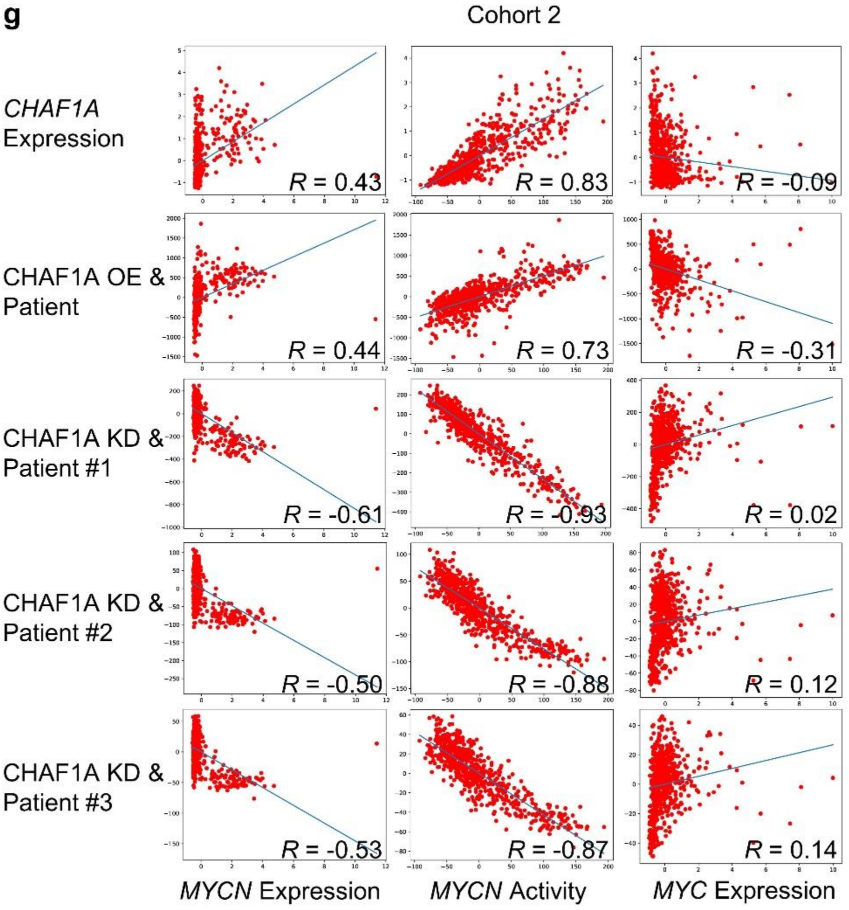
Cohort 2
CHAF1A Expression
R = 0.43
R = 0.83
R = -0.09
CHAF1A OE & Patient
R = 0.44
R = 0.73
R = -0.31
CHAF1A KD & Patient #1
R = -0.61
R = -0.93
R = 0.02
CHAF1A KD & Patient #2
R = -0.50
R = -0.88
R = 0.12
CHAF1A KD & Patient #3
R = -0.53
R = -0.87
R = 0.14
MYCN Expression
MYCN Activity
MYC Expression

Figure S7. CHAF1A/MYCN functional relationship. a) MYCN ChIP-qPCR assays in LAN5 cells. Input (white bars) and MYCN-ChIP (black bars). Samples were analyzed by qPCR using specific primers for *CHAF1A* (Table S5). Data from three independent experiments are shown (mean ± SEM, n = 3). b) mRNA expression of *CHAF1A*, *MYCN*, *MYCN* targets and polyamine genes in IMR32 cells upon CHAF1A knockdown (DOX 1 μg mL^{-1} for 0–5 days). GAPDH served as housekeeping gene. Mean ± SD (n = 3); * $p < 0.1$, **** $p < 0.0001$; two-way ANOVA with Dunnett's multiple comparisons test. c) Protein expression of CHAF1A, MYCN and ODC1 in IMR32 cells upon CHAF1A knockdown (DOX 1 μg mL^{-1} for 0–10 days). CypB served as protein loading control. Mean ± SD (n = 2–3); * $p < 0.05$, ** $p < 0.01$; one-way ANOVA with Dunnett's multiple comparisons test. d) Left, CHAF1A protein expression in IMR32 shCHAF1A cells (DOX 2 μg mL^{-1} for 5 days). GAPDH served as loading control. Right, MYCN ChIP-qPCR assays in IMR32 shCHAF1A. Input (white and grey bars) and MYCN ChIP (dark and light red bars) samples were analyzed by qPCR using specific primers for *MYCN* and *ODC1* (Table S5). Data are mean ± SEM (technical replicates n = 2). e) Left, CHAF1A and HA protein expression in IMR32 HA-CHAF1A cells (DOX 2 μg mL^{-1} for 72 h). GAPDH serves as loading control. Right, HA-CHAF1A ChIP-qPCR assays in IMR32 HA-CHAF1A cells. Input (white bars), IgG-ChIP (grey bars) and HA-ChIP (violet bars) samples were analyzed by qPCR using specific primers for *MYCN* and *ODC1* (Table S5). Data are mean ± SEM (technical replicates n = 2). f) and g) Correlation of *CHAF1A*, *MYCN* and *MYCN* signature scores in patient cohort 1 (n = 249) and cohort 2 (n = 648). Signatures are defined in the method section. Each dot indicates one patient. Correlation efficiency R is shown at bottom right corner of each graph.
